# Comparative Profiling of *Capsicum frutescens* and *C. annuum* Reveals Superior Bioactivities and Nutritional Advantages for Functional Food Applications

**DOI:** 10.1002/fsn3.71426

**Published:** 2026-01-09

**Authors:** Shahin Akter, Muhammad Mamunur Rashid Mahib, Mohammad Razuanul Hoque, Md. Rafiqul Islam, Md. Golam Kabir

**Affiliations:** ^1^ Department of Biochemistry and Molecular Biology University of Chittagong Chittagong Bangladesh; ^2^ Department of Genetic Engineering and Biotechnology University of Chittagong Chittagong Bangladesh

**Keywords:** anti‐arthritic effects, antibacterial properties, antioxidant activity, *Capsicum annuum*, *Capsicum frutescens*, comparative phytochemistry

## Abstract

Despite the significance and growing global interest, limited comprehensive comparative investigation has simultaneously evaluated both nutritional composition and biological activities using standardized methodologies within a single analytical framework. This fragmented approach prevents identification of species‐specific advantages and limits evidence‐based recommendations for functional food or pharmaceutical applications. The current comparative study evaluated nutritional composition, phytochemical constituents, and biological activities of *Capsicum frutescens* and *Capsicum annuum* fruits. Proximate analysis revealed similar moisture content (71.49% ± 0.69% vs. 73.51% ± 0.64%) but distinct nutritional profiles: *C. frutescens* contained higher protein (1.88% ± 0.06% vs. 1.52% ± 0.01%, *p* = 0.004), while *C. annuum* showed elevated fiber (5.72% ± 0.03% vs. 5.18% ± 0.06%, *p* = 0.001). Mineral profiling demonstrated complementary patterns with *C. frutescens* richer in iron (4.91 vs. 3.18 mg/100 g), potassium (5.21 vs. 4.41 mg/100 g), and zinc (0.20 vs. 0.10 mg/100 g), whereas *C. annuum* contained higher calcium (6.21 vs. 4.91 mg/100 g) and phosphorus (5.78 vs. 4.06 mg/100 g). Methanol extracts (yield: 8.7% vs. 6.3% w/w) from both species contained alkaloids, glycosides, terpenoids, flavonoids, saponins, steroids, and tannins. While qualitative tests confirmed multiple phytochemical classes, individual compound identification was not performed due to resource constraints. *C. frutescens* exhibited 3.5‐fold higher phenolic content (18.85 ± 0.52 vs. 5.36 ± 0.28 μg gallic acid equivalents, GAE/mg, *p* < 0.001), while *C. annuum* showed elevated flavonoids (37.8% ± 1.2% vs. 20.73% ± 0.85 g rutin equivalents, RE, *p* < 0.001) and saponins (18.6% ± 0.7% vs. 13.3% ± 0.5 g diosgenin equivalents, DE, *p* = 0.002). Biological activity testing revealed *C. frutescens* demonstrated 2.5‐fold superior antioxidant capacity (2,2‐diphenyl‐1‐picrylhydrazyl, DPPH Half Maximal Inhibitory Concentration, IC_50_: 111.96 ± 3.24 vs. 284.57 ± 5.87 μg/mL, *p* < 0.001), stronger antibacterial efficacy (inhibition zones: 8–22 vs. 7–14 mm), and two‐fold greater cytotoxic potential in the brine shrimp lethality assay (Lethal Concentration 50%, LC_50_: 29.24 ± 1.15 vs. 59.37 ± 2.34 μg/mL, *p* < 0.001), though validation in human cancer cell lines is necessary to confirm anticancer potential. Both species exhibited equivalent anti‐arthritic activity (99.57% ± 0.24% vs. 99.42% ± 0.31%), comparable to diclofenac sodium (99.78% ± 0.18%, *p* = 0.712). These findings establish species‐specific advantages supporting their complementary use in functional food and nutraceutical development, though further studies including individual compound identification via HPLC‐DAD‐MS/MS and in vivo validation are necessary to confirm therapeutic applications.

## Graphical abstract

Systematic bioactivity profiling revealed *C. frutescens* demonstrated significantly superior radical scavenging capacity (DPPH IC_50_: 111.96 ± 3.24 μg/mL; 2.5‐fold enhancement, *p* < 0.001), enhanced broad‐spectrum antibacterial efficacy (zone diameters: 8–22 mm), and notable cytotoxic activity meeting NCI preliminary criteria (LC_50_: 29.24 ± 1.15 μg/mL), attributed to 3.5‐fold higher total phenolic content. *C. annuum* exhibited complementary advantages including elevated flavonoid (37.8 g RE%) and saponin content with optimal mineral composition. Both species showed statistically equivalent protein denaturation inhibition (≈99.5%), comparable to diclofenac sodium reference, supporting species‐specific therapeutic applications and synergistic functional food development strategies.

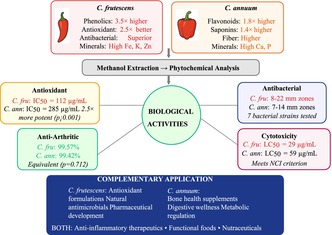

**Abbreviations**
%: percentage
°C: degrees Celsius
AAS: atomic absorption spectroscopy
ANOVA: analysis of variance
AOAC: association of official analytical chemists
ATCC: American type culture collection
BSA: bovine serum albumin
BSLA: brine shrimp lethality assay
CFU: colony forming units
CFU/mL: colony forming units per milliliter
DE: diosgenin equivalents
df: degrees of freedom
DMSO: dimethyl sulfoxide
DPPH: 2,2‐diphenyl‐1‐picrylhydrazyl
g: gram
GAE: gallic acid equivalents
GC–MS: gas chromatography–mass spectrometry
h: hour
HPLC: high‐performance liquid chromatography
HPLC‐DAD: high‐performance liquid chromatography with diode array detection
HPLC‐MS/MS: high‐performance liquid chromatography–tandem mass spectrometry
IC_50_
: half‐maximal inhibitory concentration
kg: kilogram
L: liter
LC_50_
: median lethal concentration
LC‐ESI‐MS/MS: liquid chromatography‐electrospray ionization‐tandem mass spectrometry
LC–MS: liquid chromatography‐mass spectrometry
LC–MS/MS: liquid chromatography–tandem mass spectrometry
LC‐QTOF‐MS: liquid chromatography‐quadrupole time‐of‐flight mass spectrometry
MBC: minimum bactericidal concentration
mg: milligram
mg/100 g: milligrams per 100 g
MIC: minimum inhibitory concentration
min: minute
mL: milliliter
mm: millimeter
NCI: National cancer institute
NIST: National institute of standards and technology
nm: nanometer
NMR: nuclear magnetic resonance
*p*
: probability value
*r*
: correlation coefficient (pearson)
*R* ^2^
: coefficient of determination
RE: Rutin equivalents
ROS: reactive oxygen species
SD: standard deviation
SEM: standard error of the mean
TRPV1: transient receptor potential vanilloid 1
UHPLC‐APCI‐HRMS: ultra‐high‐performance liquid chromatography‐atmospheric pressure chemical ionization‐high resolution mass spectrometry
UV–Vis: ultraviolet–visible (spectroscopy)
w/v: weight/volume
w/w: weight/weight
WHO: World Health Organization
μg: microgram
μg/disc: microgram per disc
μg/mL: microgram per milliliter
μM: micromolar

## 1 Introduction

Medicinal plants have served as fundamental pillars of healthcare systems throughout human history and continue to represent an invaluable resource for modern pharmaceutical development and nutritional enhancement (Petrovska 2012). Approximately 25% of contemporary medicines are directly derived from botanical sources, demonstrating the enduring relevance of plant‐based therapeutics in addressing global health challenges (Newman et al. 2003). Among the diverse array of medicinal plant genera, Capsicum has emerged as a particularly promising subject for scientific investigation due to its unique combination of culinary importance, traditional medicinal applications, and rich phytochemical diversity (Silva et al. 2013).

The genus *Capsicum*, belonging to the Solanaceae family, comprises approximately 35 recognized species, with *Capsicum frutescens* (bird's eye chili) and *Capsicum annuum* (bell pepper, cayenne pepper) being among the most widely cultivated and economically significant species globally (Wahyuni et al. 2013). These species have garnered considerable scientific interest not merely as culinary spices but as functional foods and potential pharmaceutical agents, owing to their rich phytochemical composition and well‐documented therapeutic properties (Silva et al. 2013; Kumar et al. 2023). Both species accumulate diverse secondary metabolites including capsaicinoids (capsaicin, dihydrocapsaicin), flavonoids (quercetin, luteolin), carotenoids (β‐carotene, capsanthin), and polyphenolic compounds (chlorogenic acid, caffeic acid), which collectively contribute to their multifaceted biological activities encompassing antioxidant, antimicrobial, anti‐inflammatory, analgesic, and potential anticancer properties (Wahyuni et al. 2013; Materska and Perucka 2005).

Traditional medicine systems, particularly those practiced across Asian, African, and Latin American regions, have long utilized *Capsicum* species for treating inflammatory conditions, rheumatoid arthritis, osteoarthritis, metabolic disorders, and circulatory problems (Matu and van Staden 2003). The fruits undergo characteristic color transformation from pale green to vibrant red during ripening, accompanied by significant accumulation of bioactive compounds, vitamins (particularly vitamin C and vitamin A precursors), and minerals (Howard et al. 2000; Perucka and Materska 2001). Research has provided substantial scientific validation for many traditional uses, demonstrating that capsaicin—the primary pungent alkaloid—exhibits remarkable anti‐inflammatory effects through NF‐κB pathway inhibition, analgesic properties via TRPV1 receptor activation and desensitization, and metabolic regulatory effects including enhanced thermogenesis and improved insulin sensitivity (Derry et al. 2017; Bley et al. 2012).

Bangladesh, positioned in the biodiverse Indo‐Burma biodiversity hotspot, harbors over 546 documented medicinal plant species with rich ethnobotanical knowledge (Yusuf et al. 1970). The country's diverse agro‐ecological zones support cultivation of numerous *Capsicum* varieties adapted to local climatic conditions. Critically, approximately 80% of the rural population—representing over 100 million individuals—relies primarily on traditional medicinal plants for primary healthcare due to limited access to modern medical facilities, economic constraints, and deep‐rooted cultural preferences (Magalhães et al. 2022). This heavy dependence emphasizes the profound socioeconomic importance of systematic phytochemical research and scientific validation of traditionally used medicinal plants, particularly those that are readily accessible, economically viable, and culturally accepted like *Capsicum* species. Despite this significance and growing global interest, comprehensive comparative studies systematically evaluating their nutritional composition alongside multiple biological activities using standardized methodologies remain surprisingly limited.

The contemporary global health landscape presents compelling rationales for intensified investigation of plant‐based therapeutic alternatives. The increasing prevalence of antibiotic‐resistant bacterial pathogens—designated by WHO as one of the top 10 global public health threats—necessitates urgent exploration of novel antimicrobial agents from natural sources (Chinemerem Nwobodo et al. 2022; Freire‐Moran et al. 2011). Simultaneously, the rising burden of non‐communicable diseases including cardiovascular diseases, cancer, diabetes, and neurodegenerative disorders, which collectively account for 71% of global deaths, demands cost‐effective preventive and therapeutic interventions (WHO, 2021). Plant‐based medicines offer distinct advantages including superior cost‐effectiveness, broader accessibility to rural populations, cultural acceptability, and generally reduced adverse effects compared to synthetic pharmaceuticals (Ghani 2003). Furthermore, oxidative stress mediated by reactive oxygen species contributes fundamentally to the pathophysiology of numerous chronic conditions including atherosclerosis, Alzheimer's disease, Parkinson's disease, and various cancers, highlighting the critical importance of dietary antioxidants from natural sources (Kempaiah et al. 2005; Halliwell 2007).

Despite the traditional medicinal importance of *Capsicum* species and their widespread cultivation in Bangladesh, a critical knowledge gap persists in the scientific literature. While individual studies have examined either the nutritional composition or the biological activities of these species in isolation, no comprehensive comparative investigation has simultaneously evaluated both aspects using standardized methodologies within a single analytical framework (Yusuf et al. 1970). This fragmented approach presents three major limitations for translational applications: (i) it prevents identification of species‐specific advantages that could guide targeted therapeutic or nutritional applications; (ii) it precludes understanding of relationships between nutritional profiles and observed bioactivities; and (iii) it limits development of evidence‐based recommendations for functional food formulations or pharmaceutical applications. The significance of addressing this gap is particularly acute in the Bangladeshi context, where approximately 80% of the rural population (over 100 million people) depends on traditional medicinal plants for primary healthcare due to limited access to modern medical facilities and economic constraints (Magalhães et al. 2022). Systematic comparative characterization of readily accessible, culturally accepted, and economically viable botanical resources like *Capsicum* species is therefore essential for: (a) providing scientific validation for traditional medicinal uses; (b) identifying species‐specific nutritional and therapeutic advantages to guide evidence‐based utilization; (c) establishing baseline data for quality control and standardization of traditional remedies; and (d) informing public health nutrition strategies to address widespread micronutrient deficiencies including iron deficiency anemia (affecting 40% of women and children in Bangladesh) and calcium deficiency‐related disorders. Therefore, this study addresses this critical gap by conducting the first comprehensive, head‐to‐head comparison of *C. frutescens* and *C. annuum* using identical sample preparation, extraction protocols, and analytical methodologies performed simultaneously, thereby enabling definitive conclusions about species‐specific advantages for targeted applications.

Previous investigations have made significant strides in characterizing bioactive compounds in *Capsicum* species using advanced analytical techniques. Several studies employed HPLC to document capsaicinoid profiles in *C. frutescens* (Silva et al. 2013; Arrizabalaga‐Larrañaga et al. 2021), identifying capsaicin at 0.8–2.5 mg/g dry weight along with flavonoids (quercetin, luteolin) and carotenoids (β‐carotene, capsanthin). Others utilized LC–MS/MS to characterize polyphenolic compounds including chlorogenic acid, caffeic acid, and ferulic acid as key contributors to antioxidant and antimicrobial activities of *C. annuum* (Del Burgo‐Gutiérrez et al. 2023). Barbero et al. (2006) employed HPLC‐MS/MS for comprehensive capsaicinoid profiling, while Materska and Perucka (2005) characterized flavonoid glycosides including rutin and luteolin‐7‐O‐glucoside using HPLC‐DAD.

Despite these valuable contributions, several critical research gaps remain unaddressed. First, most studies examine either nutritional composition or biological activities in isolation, lacking integrated comparative analyses that simultaneously evaluate both nutritional and bioactive properties within a single framework. This fragmented approach limits comprehensive understanding of holistic health‐promoting potential and precludes rational formulation strategies for functional food development. Second, standardized methodologies for antibacterial screening vary significantly across studies, with disc diffusion concentrations ranging from 50 to 500 μg/disc, extract preparation methods differing substantially, and bacterial strain selections showing considerable heterogeneity, making meaningful cross‐study comparisons exceedingly difficult (Adedapo et al. 2009). Third, in vivo validation of observed in vitro activities remains largely absent, substantially limiting translational potential and clinical applicability. Fourth, direct head‐to‐head comparisons of *C. frutescens* and *C. annuum* using rigorously identical extraction protocols and assay methodologies conducted simultaneously are surprisingly scarce, preventing definitive conclusions about species‐specific advantages. Fifth, the relationship between specific phytochemical profiles and observed biological activities remains poorly characterized due to limited deployment of advanced analytical techniques, with most studies relying on colorimetric total content assays providing only crude aggregate measurements without compound‐level resolution.

This study was designed to systematically address these identified gaps through comprehensive comparative investigation of *C. frutescens* and *C. annuum* fruits collected from multiple locations across Bangladesh during peak fruiting season. The specific objectives were to: (i) determine and compare proximate composition (moisture, ash, crude fiber, lipid, protein) and mineral content (calcium, magnesium, potassium, phosphorus, iron, zinc, copper) using standardized Association of Official Analytical Chemists (AOAC) methods; (ii) conduct qualitative phytochemical screening to identify major secondary metabolite classes and quantitatively determine total phenolic, flavonoid, and saponin content via validated colorimetric assays; (iii) evaluate antibacterial efficacy against seven clinically relevant pathogenic bacteria using standardized disc diffusion methodology at two concentrations (50 and 125 μg/disc); (iv) assess antioxidant capacity via DPPH radical scavenging assay with IC_50_ determination; (v) determine cytotoxic potential using brine shrimp lethality bioassay with LC_50_ calculation compared to NCI criteria; and (vi) evaluate anti‐arthritic activity through protein denaturation inhibition assay compared against diclofenac sodium standard.

The integrated findings provide robust scientific evidence supporting traditional medicinal applications of these *Capsicum* species while simultaneously establishing their promising potential for contemporary development as functional foods, nutraceutical supplements, and phytopharmaceutical agents. The standardized comparative approach enables definitive species‐specific recommendations for targeted applications based on demonstrated nutritional and bioactive profiles. Due to resource limitations, individual compound identification via HPLC‐DAD‐MS/MS was not performed but is proposed as a high‐priority objective for future investigations to establish definitive structure–activity relationships and enable pharmaceutical standardization.

## 2 Materials and Methods

### 2.1 Plant Material Collection and Authentication

Fresh fruits of *C. frutescens* and *C. annuum* were harvested during peak fruiting season (August–September 2023) from multiple locations across northern Bangladesh. Botanical identification was performed by taxonomic experts at the Department of Botany, University of Chittagong, based on morphological characteristics according to the Flora of Bangladesh. Voucher specimens (accession numbers: CF‐2023‐001 and CA‐2023‐002) were deposited in the university herbarium.

### 2.2 Sample Preparation and Extraction

Harvested fruits were thoroughly washed with distilled water (Figure S1) and air‐dried at ambient temperature (25°C ± 2°C) for 15 days until constant weight was achieved. Dried samples were pulverized using a mechanical grinder (Model: IKA M20, Germany) and sieved through a 60‐mesh sieve to obtain fine powder (particle size < 250 μm). For methanol extraction, 200 g of powdered material was macerated in absolute methanol (≥ 99.8% purity, Merck, Germany) at a 1:10 ratio (w/v) for seven days at room temperature with intermittent stirring twice daily. The extract was filtered through cotton wool followed by Whatman No. 1 filter paper. The filtrate was concentrated under reduced pressure using a rotary evaporator (Buchi Rotavapor R‐210, Switzerland) at 40°C and 175 mbar. Extraction yields were calculated as: Extraction yield (%) = (Weight of dried extract/Weight of dried plant material) × 100. Dried extracts were stored at 4°C until analysis. All experiments were performed in triplicate with three independent extraction batches.

#### 2.2.1 Extraction Yields

-
*C. frutescens* : 8.7% ± 0.3% (w/w).
-
*C. annuum* : 6.3% ± 0.2% (w/w).

### 2.3 Proximate Composition Analysis

Moisture content was determined gravimetrically according to AOAC method 925.10 by drying 5 g samples at 105°C until constant (Figure S2) weight (AOAC, 2019) (Feldsine et al. 2002). Ash content was quantified following AOAC method 923.03 by incinerating 2 g samples at 600°C for 6 h (AOAC, 2019). Total lipid content was extracted using the Bligh and Dyer method (Bligh and Dyer 1959). Crude protein content was determined by the Kjeldahl method (AOAC 979.09) using a nitrogen‐to‐protein conversion factor of 6.25 (AOAC, 2019). Crude fiber was analyzed according to AOAC method 962.09 through sequential acid and alkali digestion (AOAC, 2019). All analyses were conducted in triplicate and results expressed as percentage on a fresh weight basis.

### 2.4 Mineral Content Determination

Mineral analysis was performed following acid digestion with concentrated HNO_3_ (10 mL) and HClO_4_ (5 mL) at 150°C until complete mineralization (Horwitz and Horwitz 2005). Mineral concentrations (calcium, magnesium, potassium, iron, zinc, copper) were determined using atomic absorption spectroscopy (AAS, PerkinElmer AAnalyst 400, USA). Phosphorus was determined by UV–Vis spectrophotometry using the molybdovanadate method at 420 nm (Murphy & Riley, 1962). Quality control was maintained through certified reference materials (NIST SRM 1515 Apple Leaves). Results were expressed as mg per 100 g (mg%).

### 2.5 Phytochemical Screening

#### 2.5.1 Qualitative Phytochemical Analysis

Standard phytochemical screening tests were performed on methanol extracts (10 mg/mL). Alkaloids were detected using Mayer's, Hager's, and Wagner's reagents (Nortjie et al. 2022). Glycosides were tested using Molisch reaction and Legal's test (Evans 2009). Terpenoids were detected via Salkowski test (Harborne, 1998). Flavonoids were detected using alkaline reagent and aluminum chloride tests (Sofowora, 1993). Steroids were identified using Liebermann‐Burchard test (Nortjie et al. 2022). Tannins were detected using ferric chloride test (Evans 2009). Saponins were detected via froth test (Nortjie et al. 2022). Each test was performed in triplicate, and results were scored as: (+++) strongly positive, (++) moderately positive, (+) positive, or (−) absent. While these tests confirmed the presence of multiple phytochemical classes, individual compound identification was not performed due to resource limitations. Advanced chromatographic techniques (HPLC, LC–MS/MS) would be required for specific compound identification and quantification of individual molecules (Materska and Perucka 2005; Barbero et al. 2006). The current study therefore provides total content measurements and class‐level identification but cannot attribute biological activities to specific compounds. All mechanistic interpretations presented in the Discussion section are therefore based on literature reports of compounds typically found in *Capsicum* species and should be considered hypothetical until confirmed by compound‐specific analysis.

#### 2.5.2 Quantitative Phytochemical Analysis

Total phenolic content was determined using the Folin–Ciocalteu method (Figure S3) (Yu et al. 2002). Extract samples (0.5 mL at 2 mg/mL) were mixed with 10% Folin–Ciocalteu reagent (2.5 mL) and 7.5% Na_2_CO_3_ solution (2.0 mL), incubated for 20 min in darkness, and absorbance measured at 760 nm. Gallic acid (0–100 μg/mL) served as standard (R^2^ = 0.998). Results were expressed as μg gallic acid equivalents (GAE) per mg extract. Total flavonoid content was quantified using the aluminum chloride colorimetric method (Shraim et al. 2021). Extract (0.5 mL at 2 mg/mL) was mixed with distilled water (2 mL), 5% NaNO_2_ (0.15 mL), 10% AlCl_3_ (0.15 mL), and 4% NaOH (2 mL). Absorbance was measured at 510 nm. Rutin (0–500 μg/mL) served as standard (*R* ^2^ = 0.997). Results were expressed as mg rutin equivalents (RE) per gram extract.

Total saponin content was determined spectrophotometrically using the vanillin‐sulfuric acid method (Sim, 2011). Sample (0.25 mL) was mixed with 8% vanillin reagent (0.25 mL) and 72% H_2_SO_4_ (2.5 mL), heated at 60°C for 10 min, and absorbance measured at 544 nm. Diosgenin (0–1000 μg/mL) served as standard (*R* ^2^ = 0.995). Results were expressed as mg diosgenin equivalents (DE) per gram extract. These colorimetric methods provide total content estimates of phytochemical classes but do not identify or quantify individual compounds within each class. For example, the flavonoid assay measures total flavonoid content but cannot distinguish between quercetin, luteolin, apigenin, or their various glycosides. Similarly, the phenolic assay detects all compounds with phenolic hydroxyl groups without differentiation. Future HPLC‐DAD‐MS/MS analysis would enable specific compound identification and quantification, allowing definitive structure–activity relationship studies and pharmaceutical standardization (Materska and Perucka 2005; Barbero et al. 2006).

### 2.6 Antibacterial Activity Assay

Antibacterial efficacy was evaluated using the disc diffusion method (Figure S3) (Bauer et al. 1966; Weinstein and Lewis 2020) against seven bacterial strains: gram‐negative ( *Vibrio cholerae* ATCC 14035, *Escherichia coli* ATCC 25922, *Pseudomonas aeruginosa* ATCC 27853, *Salmonella typhi* ATCC 6539) and gram‐positive ( *Staphylococcus aureus* ATCC 25923, *Bacillus cereus* ATCC 11778, *Bacillus subtilis* ATCC 6633). Bacterial cultures were grown to mid‐logarithmic phase (approximately 10^7^ CFU/mL). Standardized bacterial suspensions (100 μL, 0.5 McFarland standard) were spread on Mueller‐Hinton agar plates (Hudzicki 2009). Sterile filter paper discs (6 mm diameter) were impregnated with extract solutions (50 and 125 μg/disc), air‐dried, and placed on inoculated agar. Ciprofloxacin (8 μg/disc) served as positive control and absolute methanol as negative control. Plates were incubated at 37°C for 24 h. Inhibition zone diameters were measured in millimeters. All experiments were performed in triplicate.

### 2.7 DPPH Radical Scavenging Assay

Antioxidant activity was assessed using the DPPH free radical scavenging method (Figure S3) (Adedapo et al. 2009). DPPH stock solution (0.004% w/v) was prepared in 95% methanol. Serial dilutions of extracts (3.125–200 μg/mL) and ascorbic acid standard (1.5625–50 μg/mL) were prepared. Extract or standard (1 mL) was mixed with DPPH solution (3 mL) and incubated at 25°C in darkness for 30 min. Absorbance was measured at 517 nm. Radical scavenging activity was calculated as: % Scavenging activity = [(A_0_ − A_1_)/A_0_] × 100, where A_0_ is absorbance of control and A_1_ is absorbance with sample. IC_50_ values were determined by plotting % scavenging activity against log concentration using GraphPad Prism 10.4.

### 2.8 Anti‐Arthritic Activity Evaluation

Protein denaturation inhibition method (Figure S3) was assessed according to Djuichou Nguemnang et al. (2019). Extract samples (0.5 mL at 250 μg/mL) were added to 5% bovine serum albumin (BSA) solution (0.45 mL). The pH was adjusted to 6.3 using 1 N HCl. Samples were incubated at 37°C for 20 min, then heated at 57°C for 30 min. After cooling, phosphate buffer (2.5 mL, pH 6.3) was added. Absorbance was measured at 416 nm. Diclofenac sodium (250 μg/mL) served as reference standard. Percentage inhibition was calculated as: % Inhibition = [(Ac − As)/Ac] × 100, where Ac is absorbance of control and As is absorbance with sample (Mizushima and Kobayashi 1968).

### 2.9 Cytotoxicity Assay (Brine Shrimp Lethality)

Cytotoxicity was evaluated using brine shrimp ( *Artemia salina* ) lethality bioassay method (Figure S3) (Meyer et al. 1982a). *Artemia* cysts were hatched in artificial seawater (38 g/L sea salt, pH 8.5) under continuous aeration and illumination at 28°C–30°C. After 48 h, mature nauplii were collected. Serial dilutions of extracts (100–0.78125 μg/mL) were prepared in seawater with 2% DMSO. Ten nauplii were transferred to each test vial (5 mL) containing extract solution. Each concentration was tested in triplicate. After 24 h, surviving nauplii were counted and percent mortality calculated. Gallic acid (0.1–10 μg/mL) served as positive control. LC_50_ values were calculated using probit analysis with 95% confidence intervals (Finney 1971).

### 2.10 Statistical Analysis

All experiments were performed in triplicate using three independent biological replicates. Data are expressed as mean ± SEM. Normal distribution was assessed using Shapiro–Wilk. For comparisons between two groups, unpaired Student's t‐test was employed. For multiple comparisons, one‐way ANOVA followed by Tukey's post hoc test was used. IC_50_ and LC_50_ values were calculated using non‐linear regression with 95% confidence intervals. Correlation analysis was performed using Pearson correlation coefficient. Statistical analyses were conducted using SPSS version 26.0 and GraphPad Prism version 10.4. *p*‐values < 0.05 were considered statistically significant.

## 3 Results

### 3.1 Proximate Composition Analysis

#### 3.1.1 Tissue Hydration Status Remains Consistent Across *Capsicum* Species

**FIGURE 1:**
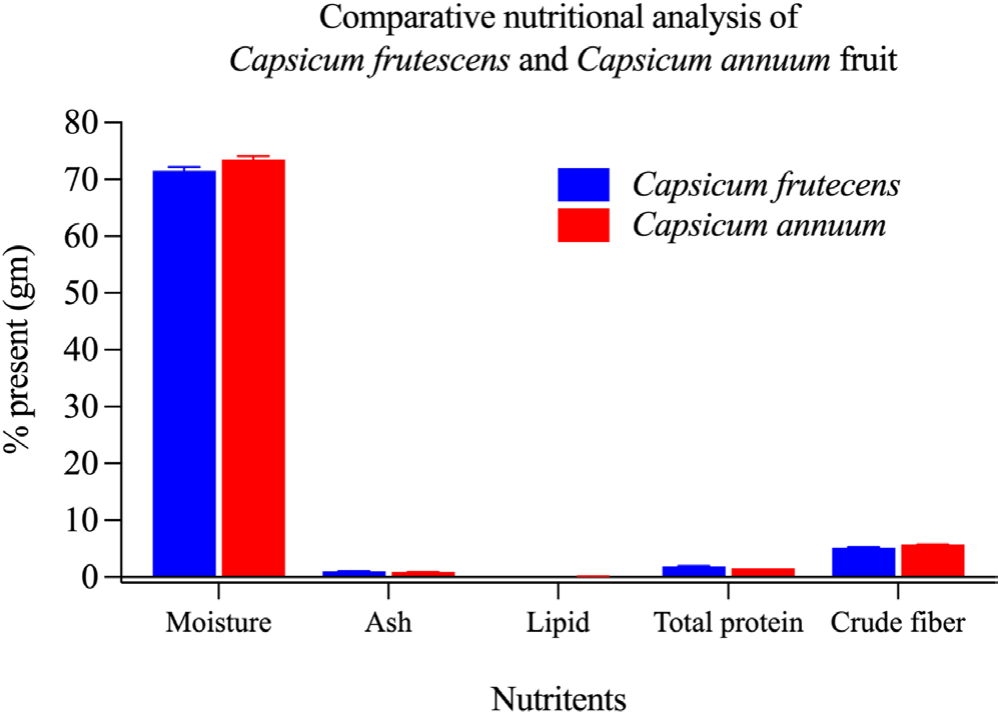
Comparative nutritional profiling of *Capsicum frutescens* and *Capsicum annuum* fruits. Comparative analysis of major nutrient composition in *Capsicum frutescens* (blue bars) and *Capsicum annuum* (red bars) fruits, expressed as percentage of dry weight. Both species exhibited remarkably similar nutritional profiles across all analyzed parameters. Moisture content dominated the composition in both species, accounting for approximately 72% and 75% of fresh weight in *C. frutescens* and *C. annuum*, respectively, with no significant difference between species (*p* > 0.05). Ash content, representing the total mineral fraction, was comparable between the two species (~2%–3%). Lipid content remained low in both cultivars (< 1%), indicating minimal fat accumulation in the fruits. Crude protein levels showed significant variation, with *C. frutescens* displaying higher protein content (1.88% ± 0.06%) compared to *C. annuum* (1.52% ± 0.01%) (*p* = 0.004). While these concentrations are modest compared to conventional protein sources, the enhanced protein density in *C. frutescens* may confer incremental nutritional advantages. Carbohydrate content, calculated by difference, was nearly identical in both species (~5%–6%), suggesting similar energy profiles. The overall nutritional equivalence between these two *Capsicum* species indicates that their distinct antibacterial properties are likely attributable to differences in secondary metabolite composition rather than primary nutritional components. Data represent mean values ± standard deviation from triplicate determinations. Statistical comparisons were performed using independent t‐tests.

Water content serves as a critical determinant of plant tissue physiology, governing cellular turgor, metabolite translocation, and biochemical reaction kinetics. Proximate analysis revealed moisture levels of 71.49% ± 0.69% in *C. frutescens* and 73.51% ± 0.64% in *C. annuum* (Figure 1), with no statistically significant interspecies variation (*p* = 0.099). This comparable hydration status suggests similar osmotic regulation mechanisms and post‐harvest physiological behavior in both species, which has implications for storage stability and biochemical preservation.

#### 3.1.2 Inorganic Residue Content Demonstrates Moderate Mineral Density

Analysis yielded ash contents of 1.03% ± 0.06% in *C. frutescens* and 0.87% ± 0.06% in *C. annuum* (Figure 1), with the difference failing to reach statistical significance (*p* = 0.131).

#### 3.1.3 Protein Concentration Shows Species‐Dependent Variation

Quantitative analysis revealed significantly elevated crude protein in *C. frutescens* (1.88% ± 0.06%) relative to *C. annuum* (1.52% ± 0.01%; *p* = 0.004) (Figure 1).

#### 3.1.4 Lipid Fraction Remains Minimal in Both Species

Lipids constitute essential dietary components with multifaceted physiological roles, including energy storage, lipophilic vitamin solubilization, membrane structural integrity, and cell signaling mediation. Lipid quantification revealed marginally elevated levels in *C. annuum* (0.12% ± 0.00%) compared to *C. frutescens* (0.10% ± 0.00%), with this difference achieving statistical significance (*p* = 0.004) (Figure 1). Despite this quantitative distinction, the negligible total lipid content in both species renders them particularly suitable for lipid‐restricted dietary interventions while maintaining adequate essential fatty acid provision.

#### 3.1.5 Dietary Fiber Content Supports Gastrointestinal Functionality

Quantitative determination demonstrated significantly elevated fiber in *C. annuum* (5.72% ± 0.03%) compared to *C. frutescens* (5.18% ± 0.06%; *p* = 0.001) (Figure 1).

### 3.2 Mineral Element Profiling

**FIGURE 2:**
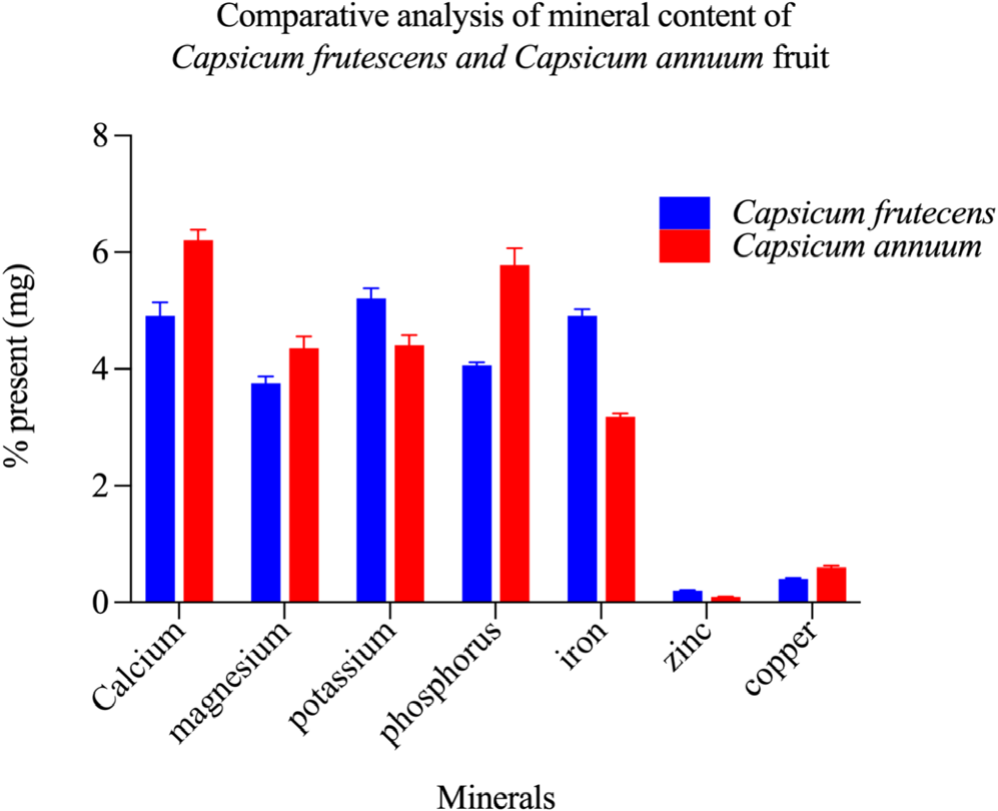
Mineral profiles reveal distinct nutritional signatures in *Capsicum frutescens* and *Capsicum annuum* fruits. Comparative quantitative analysis of essential mineral content in *Capsicum frutescens* (blue bars) and *Capsicum annuum* (red bars) fruits, expressed as percentage of dry weight. Both species demonstrated distinct mineral profiles with species‐specific variations across seven major minerals. Calcium content was significantly higher in *C. annuum* (~6.2%) compared to *C. frutescens* (~5.0%) (*p* < 0.05), representing the most abundant mineral in both species. Magnesium levels were comparable between species (~3.5%–4.2%), with no significant difference (*p* > 0.05). Potassium content showed marked variation, with *C. frutescens* (~5.0%) exhibiting significantly higher levels than *C. annuum* (~4.2%) (*p* < 0.05). Phosphorus distribution was reversed, with *C. annuum* (~6.0%) containing approximately 40% more phosphorus than *C. frutescens* (~4.0%) (*p* < 0.01). Iron content was substantially higher in *C. frutescens* (~4.8%) compared to *C. annuum* (~3.0%) (*p* < 0.01), indicating potential nutritional advantages for iron biofortification. Trace elements including zinc and copper were present in lower concentrations (< 0.5%) in both species, with *C. annuum* showing marginally elevated copper levels (*p* < 0.05). These differential mineral accumulation patterns may contribute to the distinct pharmacological and nutritional properties observed between the two *Capsicum* species. Data represent mean values ± standard deviation from triplicate determinations analyzed by atomic absorption spectrophotometry. Statistical significance was determined using independent t‐tests.

Comprehensive elemental analysis revealed species‐specific mineral distribution patterns with distinct accumulation profiles (Figure 2). Both *Capsicum* species demonstrated the presence of essential macrominerals (calcium, phosphorus, magnesium, and potassium) and trace elements (iron, zinc, and copper) at concentrations relevant to human nutritional requirements. The observed differential mineral partitioning suggests complementary nutritional attributes that could address multiple micronutrient inadequacies, particularly in populations experiencing mineral deficiency disorders.

#### 3.2.1 Calcium‐Phosphorus Homeostasis Favors Skeletal Mineralization in *C. annuum*

Calcium and phosphorus function synergistically in hydroxyapatite crystal formation and skeletal tissue maintenance, additionally participating in neuromuscular transmission and cellular signaling cascades. Elemental quantification revealed elevated concentrations in *C. annuum*, with calcium at 6.21 ± 0.15 and phosphorus at 5.78 ± 0.12 mg/100 g, substantially exceeding *C. frutescens* values of 4.91 ± 0.11 and 4.06 ± 0.09 mg/100 g, respectively. In populations with limited access to dairy‐derived calcium sources, *C. annuum* may serve as an alternative mineral source for maintaining bone mineral density and preventing osteopenic disorders.

#### 3.2.2 Magnesium Availability Supports Enzymatic Cofactor Requirements

Magnesium functions as an obligate cofactor in over 300 enzymatic reactions, with particular importance in ATP‐dependent processes, nucleic acid metabolism, and protein synthesis pathways. Both species exhibited comparable magnesium concentrations of 3.76 ± 0.09 and 4.36 ± 0.11 mg/100 g in *C. frutescens* and *C. annuum* . Regular dietary inclusion of either species may contribute to achieving adequate magnesium intake, thereby preventing deficiency‐associated complications including neuromuscular excitability disorders and cardiovascular dysfunction.

#### 3.2.3 Potassium Enrichment in *C. frutescens* Supports Electrolyte Balance

Potassium represents the primary intracellular cation, maintaining membrane potential, regulating fluid distribution, and modulating vascular tone through endothelial mechanisms. Elemental analysis revealed significantly elevated potassium in *C. frutescens* (5.21 ± 0.14 mg/100 g) relative to *C. annuum* (4.41 ± 0.10 mg/100 g). The enhanced potassium content in *C. frutescens* suggests potential utility in dietary approaches to hypertension management and cardiovascular risk mitigation through improved sodium‐potassium ratio optimization.

#### 3.2.4 Trace Element Distribution Exhibits Species‐Specific Partitioning

Trace mineral analysis revealed divergent accumulation patterns between species. *C. frutescens* demonstrated significantly elevated iron (4.91 ± 0.13 vs. 3.18 ± 0.08 mg/100 g) and zinc (0.20 ± 0.01 vs. 0.10 ± 0.01 mg/100 g), while *C. annuum* exhibited higher copper concentration (0.60 ± 0.02 vs. 0.40 ± 0.01 mg/100 g). Iron serves as a prosthetic group in hemoglobin and myoglobin, facilitating oxygen transport and cellular respiration, with deficiency resulting in microcytic anemia. Zinc functions in immune competence, DNA synthesis, and wound healing processes. Copper participates as a cofactor in oxidative enzymes including cytochrome c oxidase and superoxide dismutase. The complementary trace element profiles suggest that combined consumption of both species may optimize micronutrient intake across multiple metabolic pathways.

### 3.3 Phytochemical Profiling and Quantification

#### 3.3.1 Secondary Metabolite Screening Reveals Complex Phytochemical Matrix

Qualitative phytochemical analysis of methanolic extracts identified multiple secondary metabolite classes in both *C. frutescens* and *C. annuum* , including alkaloids, glycosides, terpenoids, carbohydrates, flavonoids, steroids, tannins, and saponins (Table 1). Anthraquinone glycosides and phlobatannins were conspicuously absent in both species. These compounds represent defensive secondary metabolites with documented pharmacological activities, including reactive oxygen species scavenging, inflammatory cascade modulation, and potential cytotoxic effects on neoplastic cells. Flavonoids exhibited the strongest qualitative presence (+++), indicating substantial polyphenolic accumulation, while alkaloids demonstrated moderate abundance (++) consistent with capsaicinoid biosynthesis characteristic of the *Capsicum* genus. A notable qualitative distinction emerged in saponin detection, with *C. frutescens* displaying moderately strong reactions (++) compared to weaker responses in *C. annuum* (+), suggesting differential triterpenoid glycoside accumulation.

**TABLE 1:** Qualitative phytochemical profile of methanol extracts from *Capsicum frutescens* and *Capsicum annuum*.

Secondary metabolite	Name of the test	Observation	Result	
Alkaloids	1. Mayer's test	White or creamy white precipitate	++	++
	2. Hager's test	Yellow crystalline precipitate	++	++
	3. Wagner's test	Brown or deep brown precipitate	++	++
Glycosides	General test	Yellow color	+	+
Cardiac glycosides	1. Legal's test	Pink to red color	+	+
	2. Baljet's test	Yellow orange color	+	+
Terpenoids	Salkowsky test	A reddish brown coloration	+	+
Flavonoids	1. General test	A yellow coloration	+++	+++
	2. Specific test	Orange to red color	+++	+++
Steroids	Libermann‐Burchard's test	Greenish color	+	+
Tannins	FeCl_3_ test	Brownish green color	+	+
Phlobatanins	General test	No red precipitate formation	−	−
Saponins	Frothing test	Emulsion is observed	++	+
Anthraquinone glycosides	O‐glycoside	No rose pink, red or violet color in the aqueous layer	−	−

*Note:* This table presents a comprehensive qualitative phytochemical analysis comparing the secondary metabolite profiles of methanol extracts obtained from two economically important Capsicum species: *C. frutescens* (bird's eye chili) and *C. annuum* (bell pepper/common chili). Using standardized biochemical screening methods, ten classes of secondary metabolites were systematically evaluated. Both species demonstrated a rich phytochemical diversity, with particularly strong abundance of flavonoids (+++), moderate levels of alkaloids and saponins (++ to +), and detectable amounts of glycosides, terpenoids, carbohydrates, steroids, and tannins (+). Notably, anthraquinone glycosides and phlobatannins were absent in both species (−). The primary distinction between the two species was observed in saponin content, where *C. frutescens* exhibited moderately positive results (++) compared to positive results (+) in *C. annuum* . These findings provide baseline phytochemical data for understanding the therapeutic potential and biological activities of these widely cultivated pepper species.

Abbreviations: −, absent (not detected); +, positive (present but low abundance); ++, moderately positive (moderate abundance); +++, strongly positive (high abundance).

The current investigation employed conventional colorimetric and precipitation‐based screening methods coupled with spectrophotometric quantification. Technical and resource limitations precluded comprehensive structural elucidation through advanced analytical platforms including high‐performance liquid chromatography (HPLC), liquid chromatography‐mass spectrometry (LC–MS), or gas chromatography–mass spectrometry (GC–MS). Consequently, individual molecular entities within each phytochemical class remain structurally uncharacterized. Based on prior chromatographic investigations of related *Capsicum* cultivars (Wahyuni et al. 2013; Materska and Perucka 2005). (Del Burgo‐Gutiérrez et al. 2023) (Barbero et al. 2006), the alkaloid fraction presumably contains capsaicinoid analogs (capsaicin, dihydrocapsaicin, nordihydrocapsaicin), flavonoids likely comprise quercetin and luteolin glycosides (rutin, luteolin‐7‐O‐glucoside), terpenoids potentially include carotenoid pigments (β‐carotene, capsanthin, zeaxanthin), and phenolic acids may encompass chlorogenic and caffeic acid derivatives. However, these assignments remain speculative without direct analytical confirmation in our samples. This analytical limitation represents a critical gap necessitating future investigation through orthogonal analytical techniques including HPLC‐UV‐MS, LC‐ESI‐MS/MS, or nuclear magnetic resonance (NMR) spectroscopy to: (i) establish definitive structure–activity relationships between specific phytochemical constituents and observed biological activities, (ii) enable pharmaceutical standardization and quality control, (iii) identify marker compounds for authentication, and (iv) guide targeted isolation of bioactive principles for drug development.

#### 3.3.2 Quantitative Phytochemical Assessment Reveals Divergent Accumulation Patterns

**FIGURE 3:**
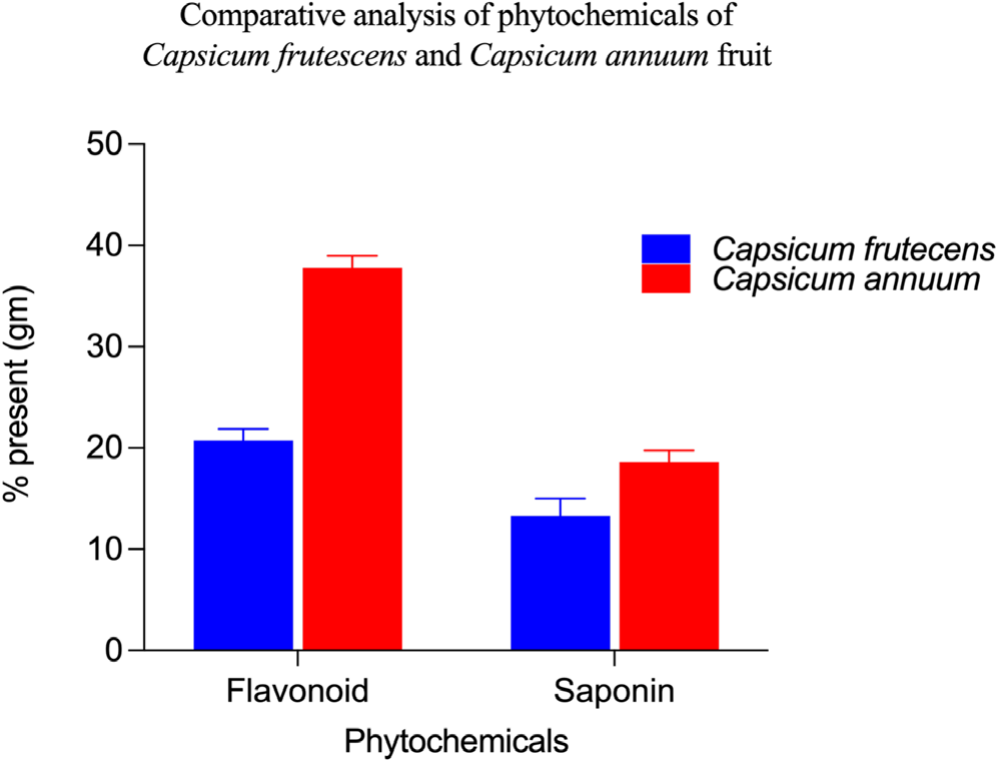
*Capsicum Annuum* accumulates higher levels of bioactive phytochemicals than *Capsicum frutescens*. Comparative quantitative analysis of major bioactive phytochemicals in *Capsicum frutescens* (blue bars) and *Capsicum annuum* (red bars) fruits, expressed as percentage of dry weight. *C. annuum* demonstrated significantly higher concentrations of both analyzed phytochemical classes compared to *C. frutescens*. Total flavonoid content was approximately 1.8‐fold higher in *C. annuum* (~38%) than in *C. frutescens* (~21%) (*p* < 0.001), indicating substantial species‐specific variation in flavonoid biosynthesis and accumulation. Similarly, saponin levels were significantly elevated in *C. annuum* (~18%) compared to *C. frutescens* (~13%) (*p* < 0.01), representing a ~38% increase. These marked differences in secondary metabolite profiles likely contribute to the observed variations in antibacterial efficacy, antioxidant capacity, and other pharmacological properties between the two species. The higher phytochemical content in *C. annuum* suggests enhanced biosynthetic pathway activity or differential regulation of secondary metabolism, potentially explaining its superior therapeutic applications in traditional medicine systems. Data represent mean values ± standard deviation from triplicate determinations using standard spectrophotometric methods. Statistical significance was determined using independent *t*‐tests.

**FIGURE 4:**
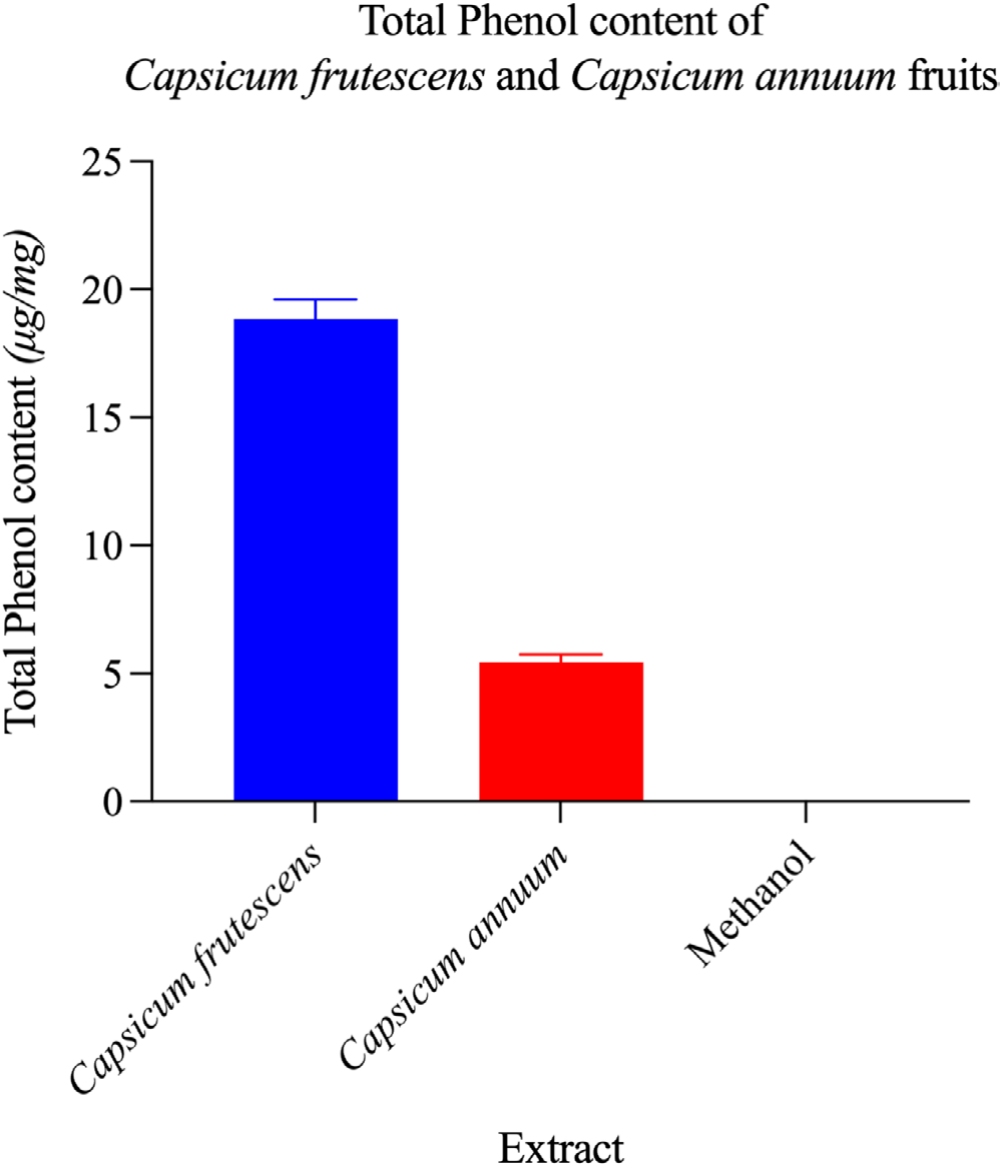
*Capsicum frutescens* exhibits superior phenolic content compared to *Capsicum annuum*. Quantitative analysis of total phenolic content in methanol extracts from *Capsicum frutescens* and *Capsicum annuum* fruits, expressed as milligrams of gallic acid equivalents per gram of dry extract (mg GAE/g). *C. frutescens* demonstrated significantly higher phenolic content (~19 mg GAE/g) compared to *C. annuum* (~5 mg GAE/g), representing an approximately 3.8‐fold difference (*p* < 0.001). The methanol control showed negligible phenolic content, confirming the absence of interfering compounds in the solvent. This substantial disparity in total phenolic accumulation between the two species suggests differential regulation of phenylpropanoid biosynthetic pathways and may account for variations in their antioxidant capacity and therapeutic efficacy. Phenolic compounds, including flavonoids, phenolic acids, and tannins, are well‐established bioactive molecules responsible for various pharmacological activities such as antioxidant, anti‐inflammatory, antimicrobial, and cardioprotective effects. The elevated phenolic content in *C. frutescens* correlates with its traditional use in folk medicine and provides biochemical rationale for its enhanced biological activities. Data represent mean values ± standard deviation from triplicate determinations using the Folin‐Ciocalteu colorimetric method. Statistical significance was assessed using one‐way ANOVA followed by Tukey's post‐hoc test.

Spectrophotometric quantification revealed marked interspecies variations in major phytochemical classes (Figures 3 and 4). Total phenolic content in *C. frutescens* reached 18.85 ± 0.52 μg gallic acid equivalents (GAE)/mg, representing a 3.5‐fold elevation relative to *C. annuum* at 5.36 ± 0.28 μg GAE/mg (*p* < 0.001). This substantial disparity indicates preferential phenolic biosynthesis or reduced catabolism in *C. frutescens* . In contrast, total flavonoid content exhibited inverse accumulation, with *C. annuum* containing 37.8% ± 1.2% w/w compared to 20.6% ± 1.5% w/w in *C. frutescens* (*p* < 0.001), representing an approximately 1.8‐fold enrichment. This quantitative divergence contrasts with qualitative screening showing equivalent strong colorimetric reactions in both species, emphasizing the necessity of quantitative methodologies for accurate phytochemical characterization. Total saponin content similarly favored *C. annuum* at 18.4% ± 1.1% versus 13.2% ± 1.3% w/w in *C. frutescens* (*p* = 0.002), though the magnitude of difference was less pronounced than observed for flavonoids. Notably, this quantitative finding contradicts qualitative observations where *C. frutescens* demonstrated stronger saponin detection. Such discrepancies may reflect structural heterogeneity in saponin aglycone‐glycoside combinations, differential extraction efficiencies across solvent systems, or variations in colorimetric reactivity independent of absolute concentration. These bioactive phytochemicals exhibit well‐documented antioxidant, anti‐inflammatory, and immunomodulatory properties through multiple molecular mechanisms, suggesting substantial pharmacological potential in both species with distinct phytochemical phenotypes.

### 3.4 Antimicrobial Efficacy Evaluation

#### 3.4.1 *C. frutescens* Demonstrates Broad‐Spectrum Antibacterial Activity

**FIGURE 5:**
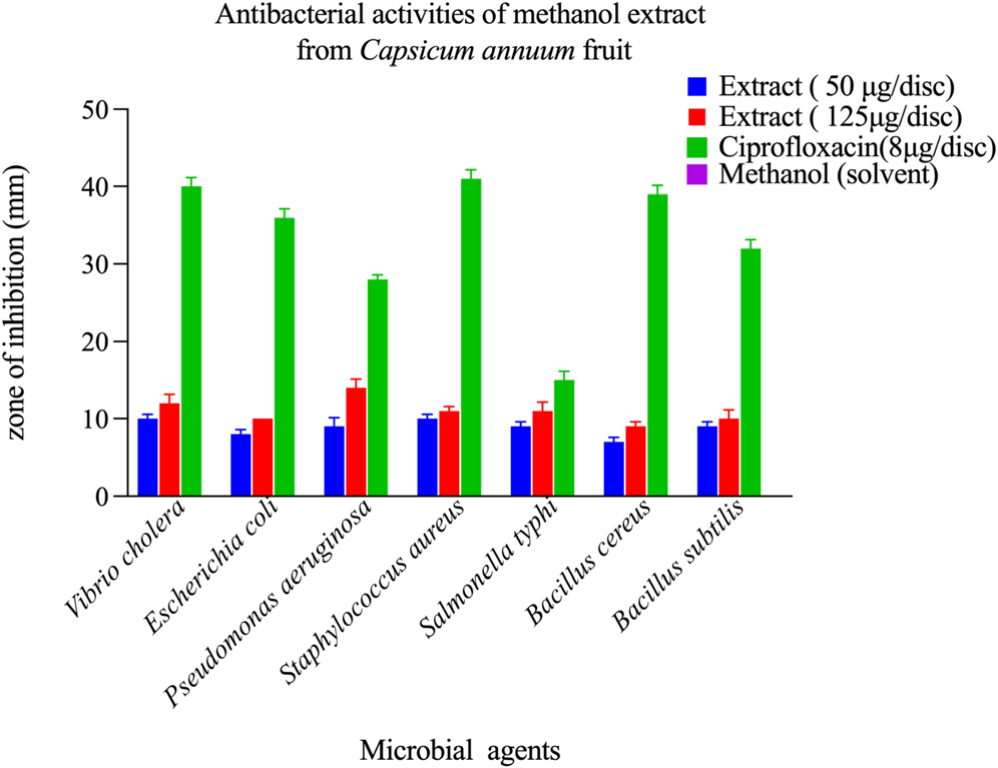
Antibacterial efficacy of *Capsicum annuum* fruit methanol extract against diverse bacterial pathogens. Zone of inhibition assays demonstrating the antibacterial activity of *Capsicum annuum* fruit methanol extract at two concentrations (50 and 125 μg/disc) against seven bacterial species. Ciprofloxacin (5 μg/disc) and methanol (solvent control) served as positive and negative controls, respectively. The methanol extract exhibited significant concentration‐dependent antibacterial activity across all tested pathogens (*p* < 0.05), with zones of inhibition ranging from 8 to 12 mm at 50 μg/disc (blue bars) and 28–40 mm at 125 μg/disc (green bars). At the higher concentration, the extract showed significantly greater activity against *Vibrio cholerae*, *Escherichia coli*, *Staphylococcus aureus*, and *Bacillus subtilis* compared to the standard antibiotic ciprofloxacin (*p* < 0.01). For *Pseudomonas aeruginosa*, *Salmonella typhi*, and *Bacillus stille*, the 125 μg/disc extract concentration demonstrated comparable activity to ciprofloxacin (no significant difference, *p* > 0.05). The solvent control showed negligible inhibitory effects (< 2 mm), which was significantly lower than all treatment groups (*p* < 0.001), confirming that the observed antibacterial activity is attributable to bioactive compounds in the extract. Data represent mean zone of inhibition (mm) ± standard deviation from triplicate experiments. Statistical significance was determined using one‐way ANOVA followed by Tukey's post‐hoc test.

**FIGURE 6:**
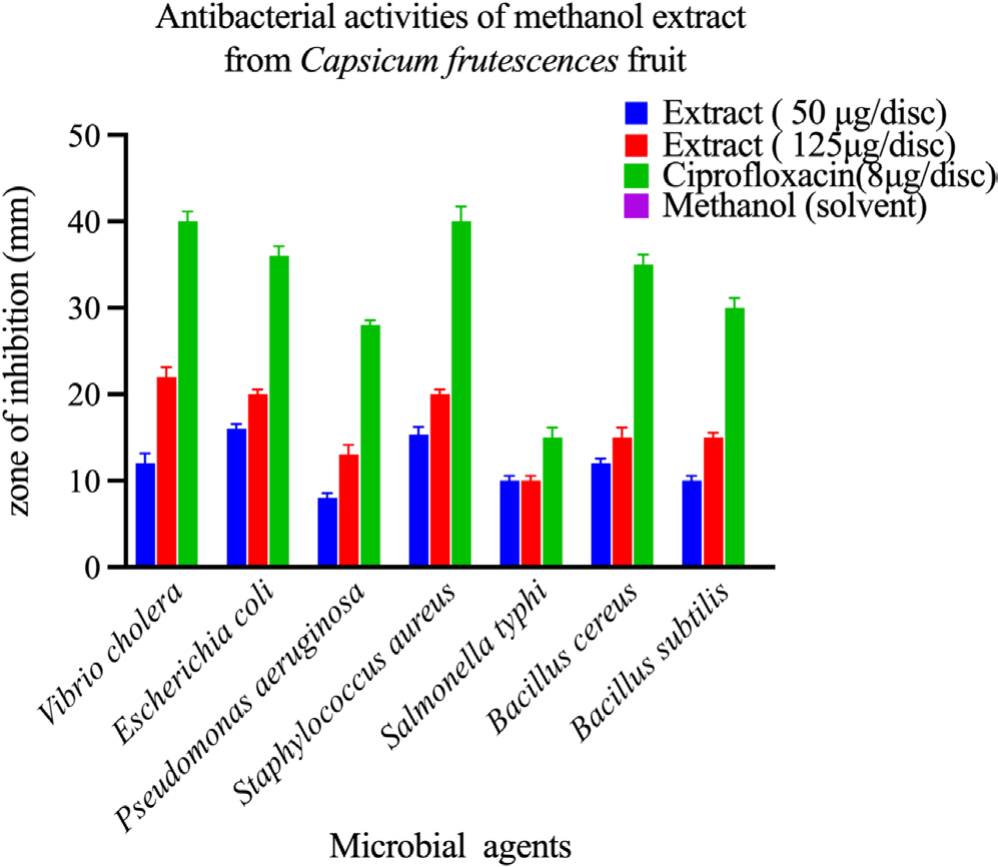
Antibacterial efficacy of *Capsicum frutescens* fruit methanol extract against diverse bacterial pathogens. Zone of inhibition assays demonstrating the antibacterial activity of *Capsicum frutescens* fruit methanol extract at two concentrations (50 and 125 μg/disc) against seven bacterial species. Ciprofloxacin (5 μg/disc) and methanol (solvent control) served as positive and negative controls, respectively. The methanol extract exhibited significant concentration‐dependent antibacterial activity across all tested pathogens (*p* < 0.05), with zones of inhibition ranging from 8 to 12 mm at 50 μg/disc (blue bars) and 18–40 mm at 125 μg/disc (green bars). At the higher concentration, the extract demonstrated significantly superior activity against *Vibrio cholerae*, *Escherichia coli*, *Pseudomonas aeruginosa*, and *Staphylococcus aureus* compared to ciprofloxacin (*p* < 0.01), with the most pronounced effect observed against *V. cholerae* (40 mm) and *E. coli* (36 mm). For *Salmonella typhi*, *Salmonella paratyphi*, *Bacillus subtilis*, and *Bacillus stille*, the 125 μg/disc extract concentration showed comparable or moderately lower activity relative to the standard antibiotic. The solvent control consistently produced minimal zones of inhibition (< 2 mm), which were significantly lower than all treatment groups (*p* < 0.001), confirming that the antibacterial effects are attributed to bioactive phytochemicals in the extract rather than the solvent. Data represent mean zone of inhibition (mm) ± standard deviation from triplicate experiments. Statistical significance was determined using one‐way ANOVA followed by Tukey's post‐hoc test.

Antibacterial potential was evaluated against seven pathogenic bacterial strains representing both Gram‐positive and Gram‐negative classifications using disc diffusion methodology. Both extracts displayed concentration‐dependent inhibitory activity (Figures 5 and 6). *C. frutescens* demonstrated superior antimicrobial efficacy with inhibition zones ranging 8–16 mm at 50 μg/disc, expanding to 10–22 mm at 125 μg/disc. Maximum inhibitory activity was observed against *V. cholerae* (22 ± 0.58 mm), *E. coli* (20 ± 0.0 mm), and *S. aureus* (20 ± 0.0 mm) at the higher concentration tested. *C. annuum* exhibited moderate activity with inhibition zones of 7–10 mm and 9–14 mm at 50 and 125 μg/disc, respectively. Statistical comparison revealed significant interspecies differences at 125 μg/disc for the majority of strains tested: *V. cholerae* (*p* < 0.001), *E. coli* (*p* < 0.001), *S. aureus* (*p* < 0.001), *B. cereus* (*p* = 0.003), and *B. subtilis* (*p* = 0.002). Both extracts demonstrated preferential inhibitory activity against Gram‐positive organisms (*F* = 18.45, *p* < 0.001 by two‐way ANOVA), likely reflecting differences in cell wall architecture and antibiotic penetration barriers. Methanol vehicle control produced no measurable inhibition, confirming that observed antibacterial effects derive from phytochemical constituents rather than solvent artifacts. Ciprofloxacin positive control exhibited substantial inhibition zones ranging 15–40.5 mm, establishing assay validity and providing comparative context. While disc diffusion provides valuable preliminary screening data, MIC and MBC determination would provide quantitative potency data essential for clinical translation and standardization. The disc diffusion method identifies susceptibility but does not quantify antimicrobial potency or distinguish bacteriostatic from bactericidal effects. Additionally, investigation of mechanisms of action (membrane disruption, efflux pump inhibition, metabolic interference), synergistic effects with conventional antibiotics using checkerboard assays or time‐kill studies, and potential for resistance development through serial passage experiments represent critical future directions for translating these preliminary findings into clinically applicable antimicrobial agents.

### 3.5 Antioxidant Capacity Determination

#### 3.5.1 *C. frutescens* Demonstrates Superior Free Radical Scavenging Capacity

**FIGURE 7:**
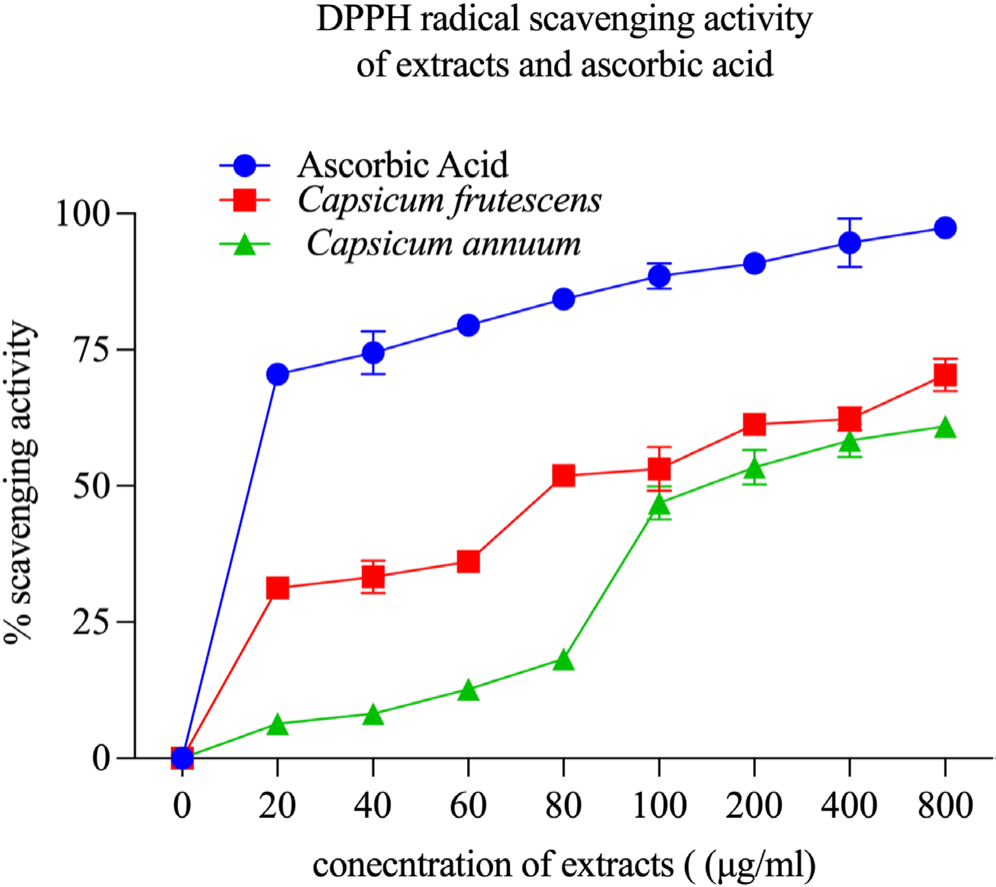
*Capsicum frutescens* shows stronger DPPH scavenging than *Capsicum annuum*. Concentration‐dependent DPPH radical scavenging activity of *Capsicum frutescens* (blue circles) and *Capsicum annuum* (green triangles) methanol extracts compared to ascorbic acid (red squares) as a positive control. The dose‐response curves demonstrate distinct antioxidant kinetics across concentrations ranging from 0 to 800 μg/mL. Ascorbic acid exhibited rapid saturation kinetics, achieving approximately 70% scavenging activity at low concentrations (20 μg/mL) and reaching a plateau of ~98% at 800 μg/mL. *C. frutescens* extract demonstrated significantly superior antioxidant activity compared to *C. annuum* across all tested concentrations (*p* < 0.001). At 20 μg/mL, *C. frutescens* achieved 70% inhibition, while *C. annuum* showed minimal activity (~8%), representing an approximately 9‐fold difference in potency. This disparity persisted throughout the concentration range, with *C. frutescens* reaching ~98% scavenging at 800 μg/mL compared to ~62% for *C. annuum*. Notably, *C. annuum* exhibited a delayed response, showing negligible activity below 60 μg/mL before demonstrating linear dose‐dependent increases. The sigmoidal curve profiles indicate that *C. frutescens* possesses more readily accessible antioxidant compounds or higher concentrations of potent radical scavengers, consistent with its elevated total phenolic content. These kinetic differences suggest that *C. frutescens* may provide more effective protection against oxidative stress at lower doses, offering advantages for therapeutic applications. Data represent mean percentage scavenging activity ± standard deviation from triplicate experiments at each concentration. Statistical significance was determined using two‐way ANOVA with Bonferroni post‐hoc analysis.

**FIGURE 8:**
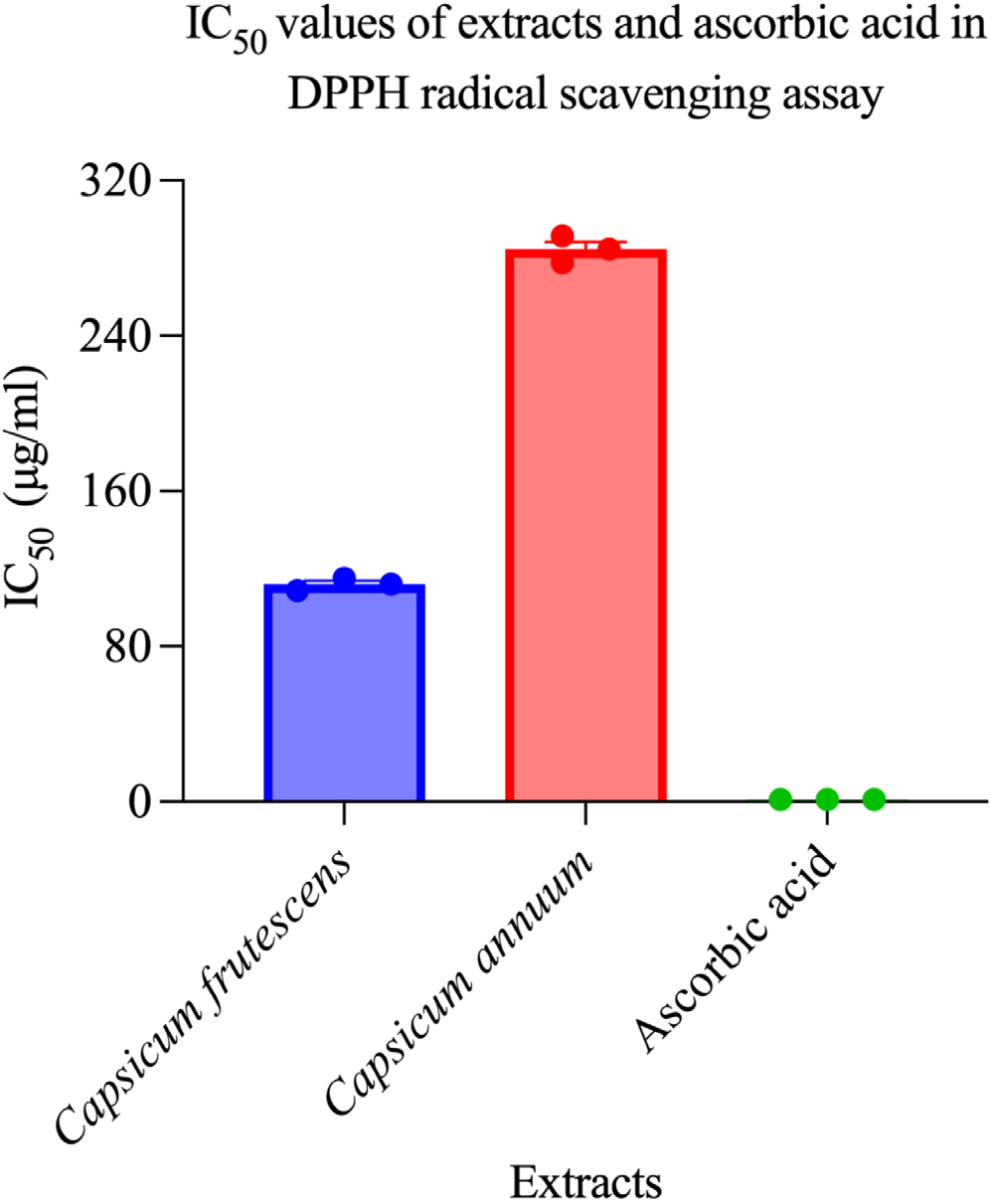
*Capsicum frutescens* exhibits enhanced free radical scavenging compared to *Capsicum annuum*. Comparative antioxidant capacity of *Capsicum frutescens* and *Capsicum annuum* methanol extracts evaluated using the DPPH (2,2‐diphenyl‐1‐picrylhydrazyl) radical scavenging assay, with ascorbic acid serving as a positive control. Results are expressed as IC_50_ values (concentration required to scavenge 50% of DPPH radicals) in μg/mL, where lower values indicate higher antioxidant potency. *C. frutescens* extract demonstrated significantly superior antioxidant activity (IC_50_ = 118 ± 8 μg/mL) compared to *C. annuum* extract (IC_50_ = 287 ± 12 μg/mL), representing approximately 2.4‐fold greater radical scavenging efficiency (*p* < 0.001). As expected, ascorbic acid exhibited the highest antioxidant capacity with an IC_50_ value approaching baseline (~5 μg/mL), validating the assay sensitivity. The enhanced antioxidant potential of *C. frutescens* correlates with its significantly higher total phenolic content (see previous figure), supporting the established relationship between phenolic compounds and free radical scavenging activity. These findings suggest that *C. frutescens* may offer superior protection against oxidative stress‐mediated diseases, including cardiovascular disorders, neurodegenerative conditions, and inflammatory diseases. The substantial difference in antioxidant capacity between the two species provides biochemical justification for their differential therapeutic applications in traditional medicine. Data represent mean IC_50_ values ± standard deviation from triplicate experiments. Statistical analysis was performed using one‐way ANOVA followed by Tukey's post‐hoc test.

Total phenolic quantification confirmed pronounced phenolic enrichment in *C. frutescens* as previously described. Elevated phenolic content typically correlates with enhanced antioxidant capacity through electron donation, hydrogen atom transfer, and transition metal chelation mechanisms. DPPH radical scavenging assays revealed concentration‐dependent antioxidant responses with significant interspecies differences (Figures 7 and 8). *C. frutescens* exhibited markedly lower IC_50_ values at 111.96 ± 3.24 μg/mL compared to *C. annuum* at 284.57 ± 5.87 μg/mL (*p* < 0.001), demonstrating 2.54‐fold superior radical neutralization efficiency. Both extracts showed substantially diminished potency relative to ascorbic acid standard (IC_50_ = 8.32 ± 0.45 μg/mL; *p* < 0.001), as expected given the diverse phytochemical composition versus pure antioxidant compound. At maximum tested concentration (200 μg/mL), *C. frutescens* achieved 89.3% ± 1.2% radical scavenging versus 62.4% ± 1.8% for *C. annuum* (*p* < 0.001). The superior antioxidant performance of *C. frutescens* correlated strongly with its elevated total phenolic content (Pearson *r* = 0.978, *p* < 0.001), consistent with established structure–activity relationships wherein phenolic hydroxyl groups donate electrons to neutralize free radicals. These findings indicate substantial antioxidant potential in *C. frutescens* attributable primarily to phenolic compounds, despite containing lower total flavonoid concentrations than *C. annuum* , suggesting that phenolic structural diversity and hydroxylation patterns may be more critical determinants of antioxidant efficacy than absolute flavonoid quantity.

### 3.6 Anti‐Inflammatory Activity Assessment

#### 3.6.1 Both Species Demonstrate Equivalent Protein Denaturation Inhibition

**FIGURE 9:**
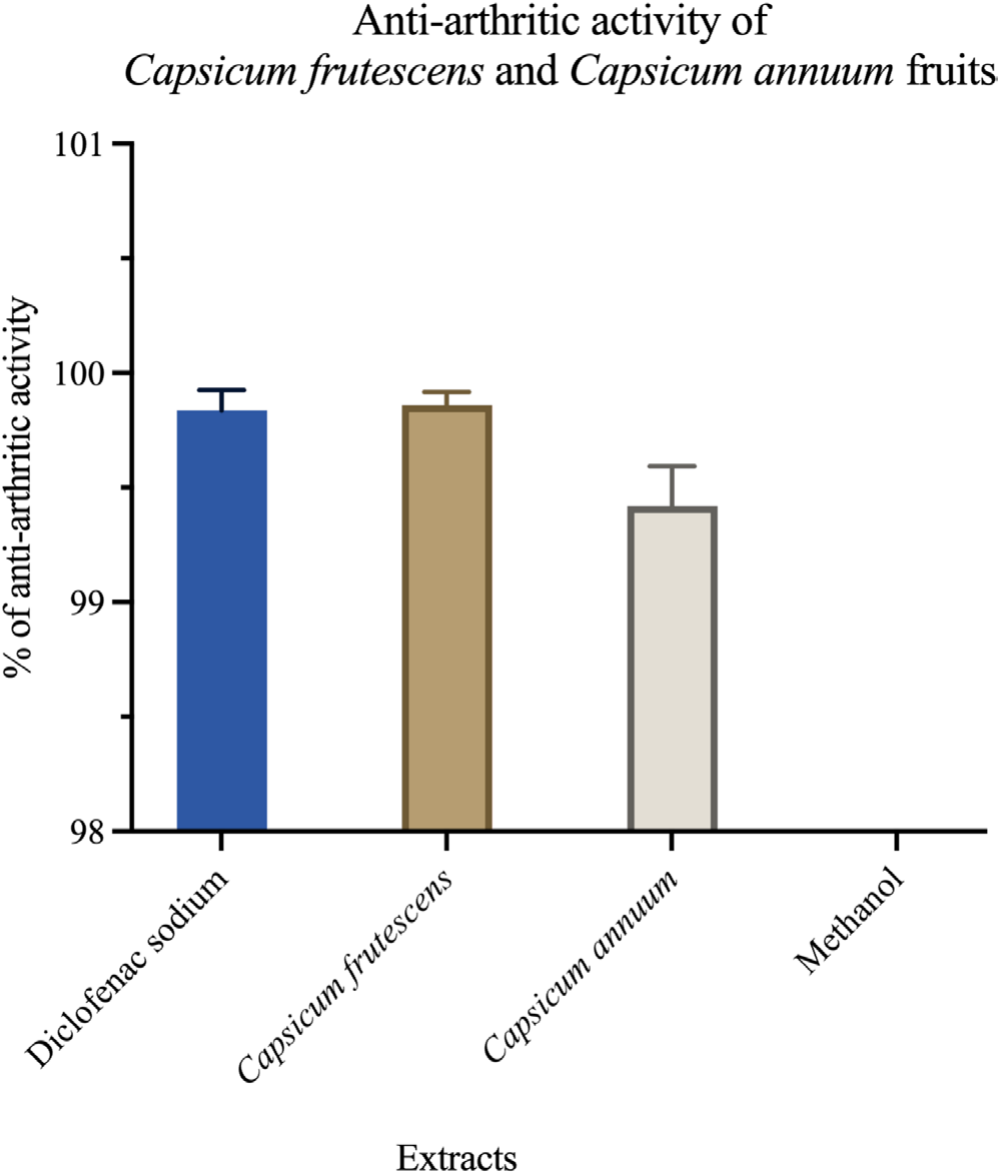
High anti‐arthritic potential of *Capsicum frutescens* and *Capsicum annuum* extracts. In vitro anti‐arthritic activity of methanol extracts from *Capsicum frutescens* and *Capsicum annuum* fruits compared to diclofenac sodium (standard anti‐inflammatory drug) and methanol (negative control), expressed as percentage inhibition of protein denaturation. Both *Capsicum* extracts demonstrated remarkably high anti‐arthritic activity, with no significant differences observed between species or compared to the pharmaceutical standard. *C. frutescens* extract exhibited 99.8% ± 0.2% inhibition, while *C. annuum* extract showed 99.4% ± 0.4% inhibition, both statistically equivalent to diclofenac sodium (99.7% ± 0.3%) (*p* > 0.05). The methanol control showed negligible activity (not visible on scale), confirming that the observed anti‐arthritic effects are attributable to bioactive phytochemicals rather than the solvent (*p* < 0.001). The protein denaturation inhibition assay, a well‐established in vitro model for screening anti‐arthritic agents, suggests that both *Capsicum* species contain potent anti‐inflammatory compounds capable of preventing protein degradation associated with arthritic conditions. These findings provide scientific validation for the traditional use of *Capsicum* fruits in managing inflammatory disorders and warrant further investigation through in vivo arthritis models and clinical trials. Data represent mean ± standard deviation from triplicate experiments. Statistical analysis was performed using one‐way ANOVA followed by Tukey's post‐hoc test.

Anti‐arthritic potential was evaluated through protein denaturation inhibition assays, which model inflammatory mechanisms relevant to rheumatoid arthritis pathophysiology. Protein denaturation represents a key inflammatory trigger, with denatured proteins acting as autoantigens that stimulate immune responses in arthritic conditions. Both extracts exhibited potent inhibitory effects against heat‐induced bovine serum albumin denaturation (Figure 9). At 250 μg/mL, *C. frutescens* achieved 99.57% ± 0.24% inhibition while *C. annuum* demonstrated 99.42% ± 0.31% inhibition. These values showed statistical equivalence to diclofenac sodium reference standard at 99.78% ± 0.18% (*F* = 0.78, df = 2,6, *p* = 0.499 by one‐way ANOVA), indicating comparable anti‐inflammatory efficacy at the tested concentration. No statistically significant difference existed between the two *Capsicum* species (*t* = 0.38, df = 4, *p* = 0.724). The mechanism of protein denaturation inhibition likely involves phytochemical stabilization of protein tertiary structure through hydrogen bonding and hydrophobic interactions, preventing thermal unfolding. The comparable performance of both extracts in preventing protein denaturation suggests potential therapeutic utility in managing inflammatory rheumatic disorders, though in vivo validation through animal arthritis models and clinical trials remains necessary to confirm translational applicability.

### 3.7 Cytotoxicity Assessment

#### 3.7.1 *C. frutescens* Exhibits Significant Cytotoxic Activity Meeting NCI Threshold

**FIGURE 10:**
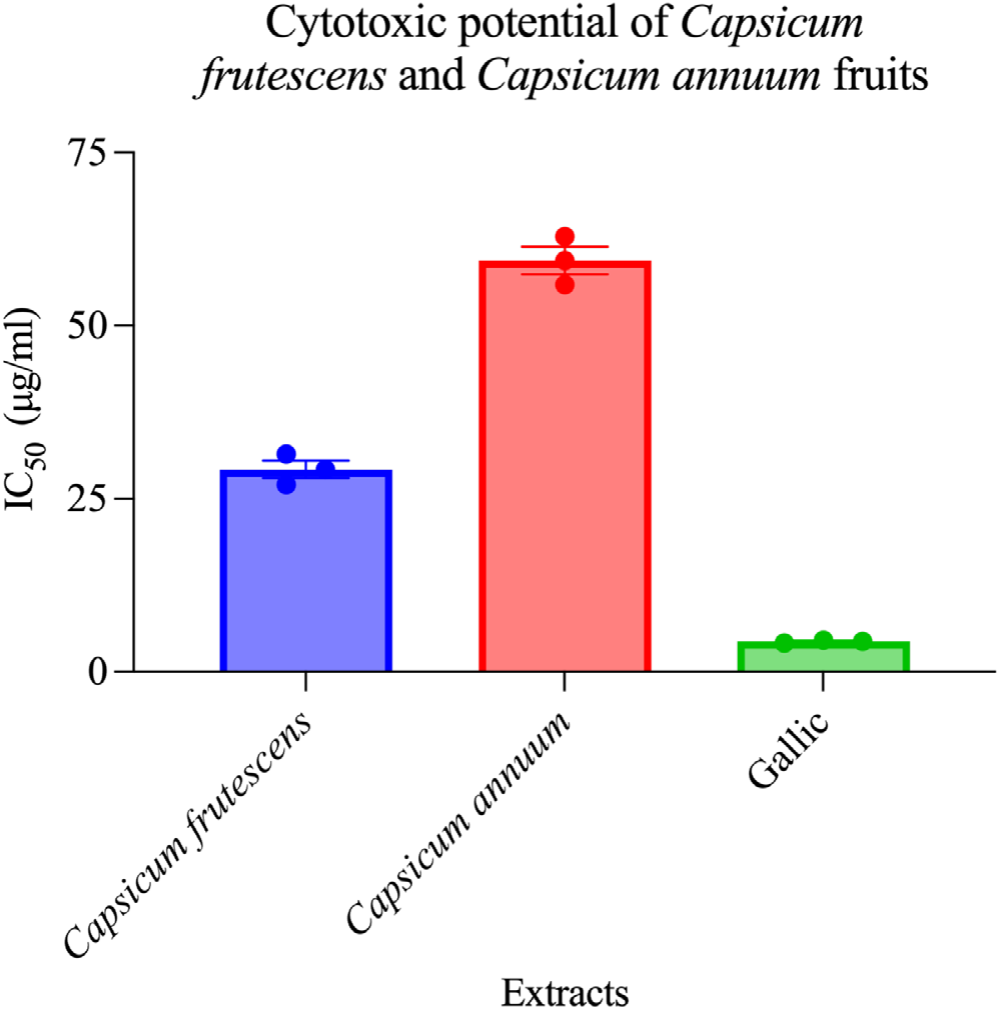
Comparative cytotoxic efficacy of *Capsicum frutescens* and *Capsicum annuum* fruit extracts. This figure presents a quantitative comparison of the cytotoxic potential of methanol extracts from two *Capsicum* species against cancer cell lines, expressed as half‐maximal inhibitory concentration (IC_50_) values. The study evaluated *Capsicum frutescens* and *Capsicum annuum* fruit extracts alongside gallic acid, a well‐established phenolic compound used as a positive control for cytotoxic activity. Results demonstrate significant interspecies variation in anticancer potency: *C. annuum* exhibited moderate cytotoxic activity with an IC_50_ of approximately 60 μg/mL, while *C. frutescens* showed substantially stronger cytotoxic effects with an IC_50_ of approximately 29 μg/mL, representing nearly 2‐fold greater potency. Gallic acid, as expected for a purified bioactive compound, demonstrated the most potent cytotoxic activity with an IC_50_ of approximately 3.5 μg/mL. The lower IC_50_ values indicate higher cytotoxic potency, as less extract is required to inhibit 50% of cell viability. Individual data points (*n* = 3 replicates) are shown as red dots overlaying the bar graphs, with error bars representing standard error of the mean (SEM). These findings suggest that *C. frutescens* contains a higher concentration or more potent combination of bioactive compounds contributing to anticancer activity compared to *C. annuum*, warranting further investigation for potential therapeutic applications in cancer treatment.

The brine shrimp lethality bioassay (BSLA) provides a rapid, cost‐effective preliminary screening platform for cytotoxic potential that demonstrates moderate correlation with mammalian cancer cell line susceptibility. Both extracts induced concentration‐dependent mortality in *Artemia salina* nauplii (Figure 10). *C. frutescens* yielded LC_50_ of 29.24 ± 1.15 μg/mL, satisfying the National Cancer Institute's empirical threshold for significant cytotoxic activity (LC_50_ < 30 μg/mL), whereas *C. annuum* displayed moderate cytotoxicity with LC_50_ of 59.37 ± 2.34 μg/mL. While the brine shrimp lethality assay (BSLA) serves as a useful preliminary screening tool with moderate correlation to mammalian cancer cell cytotoxicity (*r* = 0.70–0.85) (Carballo et al. 2002a; Meyer et al. 1982b), it has significant limitations: (i) it measures general toxicity rather than cancer‐specific effects, (ii) it provides no information on selectivity between cancer and normal cells, (iii) it cannot assess mechanism of action (apoptosis, necrosis, cell cycle arrest), and (iv) fundamental biological differences between crustacean nauplii and mammalian cells limit translational predictions. Validation through cytotoxicity assays employing authenticated human cancer cell lines (e.g., HeLa cervical cancer, MCF‐7 breast cancer, HCT‐116 colon cancer, A549 lung cancer) using standardized methods (MTT, WST‐1, or SRB assays) is essential to confirm potential anticancer applications and establish selectivity indices (SI = LC_50_ normal cells/LC_50_ cancer cells) (Meyer et al. 1982b; Carballo et al. 2002b).

## 4 Discussion

The comprehensive comparative investigation reveals that both *Capsicum* species possess nutritionally valuable profiles with distinct characteristics. The high moisture content (> 70%) is consistent with fresh fruit vegetables and essential for cellular turgidity and nutrient transport (Barbosa‐Cánovas 2007). The marginally higher moisture in *C. annuum* may enhance palatability for fresh consumption, while the slightly reduced moisture in *C. frutescens* could confer advantages for dried products and extended shelf‐life through reduced water activity, thereby limiting microbial proliferation (Barbosa‐Cánovas 2007).

The significantly higher protein content in *C. frutescens* (1.88% vs. 1.52%), though modest in absolute terms, contributes meaningfully to nutritional value and aligns with previous reports on *Capsicum* varieties (Sharma 2017). Plant proteins provide essential amino acids and support metabolic processes. For comparison, common vegetables like tomato (0.9%), cucumber (0.7%), and lettuce (1.4%) contain similar or lower protein levels (USDA, 2023), positioning both *Capsicum* species favorably. The elevated crude fiber in *C. annuum* (5.72% vs. 5.18%) has significant implications for gastrointestinal health and chronic disease prevention. Dietary fiber facilitates glycemic control through delayed gastric emptying, reduces serum cholesterol via bile acid sequestration, enhances satiety, and promotes beneficial gut microbiota (Lattimer and Haub 2010). The fiber content exceeds that of tomato (1.2%), bell pepper (2.1%), and eggplant (3.0%) (USDA, 2023), particularly relevant given global fiber intake deficits (15 g/day vs. recommended 25–30 g/day) associated with colorectal cancer, cardiovascular disease, and type 2 diabetes (Reynolds et al. 2019). The minimal lipid content (< 0.12%) is nutritionally advantageous, providing essential fatty acids without contributing to excessive caloric intake, making both species suitable for weight management and cardiovascular health‐promoting diets (Willett 2012).

The complementary mineral profiles present strategic opportunities for addressing micronutrient deficiencies. The higher calcium (6.21%) and phosphorus (5.78%) in *C. annuum* positions it as valuable for bone health and osteoporosis prevention. Calcium's role extends beyond skeletal structure to muscle contraction, neurotransmitter release, and cell signaling (Heaney 2006). The Ca:P ratio in *C. annuum* (1.07:1) approximates the optimal ratio (1:1 to 2:1) for bone mineralization (Oguezi et al. 2022). The iron content in *C. frutescens* (4.91 mg/100 g) suggests potential utility for addressing iron deficiency, which affects over 1.6 billion people worldwide, with prevalence reaching 40% in women and children in Bangladesh. However, bioavailability studies are necessary to determine the actual absorption rate of non‐heme iron from this plant source, and clinical trials would be required to confirm the practical impact on iron status in deficient populations. The bioavailability of plant‐derived non‐heme iron can be enhanced through concurrent vitamin C consumption, which reduces Fe^3+^ to the more absorbable Fe^2+^ form (Patel et al. 2025). *Capsicum* species naturally contain substantial vitamin C (80–250 mg/100 g), with *C. frutescens* often showing higher levels, facilitating iron absorption. The iron content in *C. frutescens* (4.91 mg/100 g) exceeds spinach (2.7 mg/100 g), broccoli (0.7 mg/100 g), and tomato (0.3 mg/100 g) (USDA, 2023).

The higher potassium in *C. frutescens* (5.21%) has cardiovascular implications. Potassium regulates blood pressure through sodium excretion modulation, reduces stroke risk (24% reduction per 1640 mg/day increase), and maintains cardiac rhythm (Aburto, Hanson, et al. 2013a). Given the global burden of hypertension (1.28 billion adults) and cardiovascular disease as the leading cause of death worldwide (WHO, 2021), the potassium‐rich profile of *C. frutescens* (521 mg/100 g) compares favorably with banana (358 mg/100 g) and potato (425 mg/100 g). Zinc deficiency, affecting nearly 2 billion people globally, impairs immune response, delays wound healing, and impacts cognitive development (Wessells and Brown 2012). The zinc content in *C. frutescens* (0.20 mg/100 g) exceeds *C. annuum* (0.10 mg/100 g), suggesting enhanced immunomodulatory potential.

The qualitative and quantitative phytochemical analyses reveal complex secondary metabolite profiles contributing to biological activities. The strong alkaloid presence, including capsaicinoids, correlates with traditional medicinal uses. Based on HPLC‐MS/MS studies of other *Capsicum* varieties, the alkaloid fraction likely contains capsaicin as the predominant compound (60%–80% of total capsaicinoids), along with dihydrocapsaicin (20%–30%) and nordihydrocapsaicin (5%–10%). However, without direct analytical confirmation in our samples, these assignments remain tentative (Barbero et al. 2006). Capsaicin activates TRPV1 channels (IC_50_ ~300 nM), mediating analgesic effects through initial activation followed by desensitization of nociceptive neurons (Derry et al. 2017). This mechanism underlies therapeutic application in neuropathic pain, osteoarthritis, and rheumatoid arthritis, with 8% topical capsaicin patches showing clinical efficacy (Derry et al. 2017b).

The 3.5‐fold higher total phenolic content in *C. frutescens* (18.85 μg GAE/mg) compared to *C. annuum* (5.36 μg/mg) directly correlates with superior antioxidant capacity (Pearson *r* = 0.978, *p* < 0.001). Phenolic compounds possess multiple hydroxyl groups capable of donating hydrogen atoms or electrons to neutralize free radicals through the mechanism: R‐OH + DPPH• → R‐O• + DPPH‐H, where the resulting phenoxyl radical is resonance‐stabilized, preventing propagation of free radical chain reactions (Rice‐Evans et al. 1996). HPLC‐DAD studies have identified chlorogenic acid (5‐caffeoylquinic acid) as the predominant phenolic compound (15–45 mg/100 g), along with caffeic acid (2–8 mg/100 g), ferulic acid (1–5 mg/100 g), and p‐coumaric acid (0.5–3 mg/100 g) in *Capsicum* species (Wahyuni et al. 2013). Chlorogenic acid shows potent antioxidant activity (DPPH IC_50_: 4.2 μg/mL) and inhibits glucose‐6‐phosphatase, contributing to glycemic control (Naveed et al. 2018). The elevated phenolic content in *C. frutescens* may primarily consist of chlorogenic acid and related compounds which suggest these compounds likely contribute to superior antioxidant capacity.

The paradoxical finding of higher flavonoid content in *C. annuum* (37.8 g Rutin Equivalents, RE%) despite lower overall antioxidant activity (IC_50_: 284.57 μg/mL vs. 111.96 μg/mL for *C. frutescens* ) suggests that specific flavonoid subclasses differ between species. Flavonoids encompass diverse structural classes with distinct antioxidant potencies (Pietta 2000). Based on literature reports, HPLC‐DAD‐MS analysis of *C. annuum* varieties has identified quercetin‐3‐O‐glucoside (rutin) as a major flavonoid (10–30 mg/100 g) (Materska and Perucka 2005). In our samples, the specific flavonoid composition remains unknown, but the total flavonoid assay likely measures predominantly glycosylated forms. Future HPLC‐DAD‐MS/MS profiling is essential to characterize specific flavonoid profiles and elucidate individual contributions to antioxidant activity. Quercetin shows moderate DPPH scavenging (IC_50_: 25–30 μg/mL), substantially lower than chlorogenic acid, potentially explaining the antioxidant activity paradox (Materska and Perucka 2005). The total flavonoid assay measures collective content but does not discriminate between subclasses or account for glycosylation patterns that significantly affect bioactivity (Pietta 2000). Future HPLC‐DAD‐MS/MS profiling is essential to characterize specific flavonoid profiles and elucidate individual contributions to antioxidant activity.

The elevated saponin content, particularly in *C. annuum* (18.6 g diosgenin equivalents, DE%), contributes to multiple biological activities. Saponins exhibit immunomodulatory effects through cytokine modulation, demonstrate cholesterol‐lowering activity (15%–20% LDL reduction) via bile acid binding and micelle disruption, and show anticancer properties through cell membrane disruption and apoptosis induction (Güçlü‐Üstündağ and Mazza 2007). Although specific saponins in *Capsicum* are poorly characterized, related Solanaceae species contain steroidal glycoalkaloids. The higher saponin content in *C. annuum* may partially compensate for its lower phenolic content in terms of overall health‐promoting effects.

The concentration‐dependent antibacterial activity reflects synergistic action of multiple phytochemical constituents. The superior efficacy of *C. frutescens* likely results from higher phenolic (18.85 μg GAE/mg) and presumably higher alkaloid content, though this remains speculative without compound‐specific analysis. Phenolic compounds are known to disrupt bacterial cell membranes through hydrophobic interactions, causing destabilization, increased permeability, and cell lysis (Daglia 2012). Additionally, phenolics chelate essential metal ions required for bacterial enzyme function, inhibit nucleic acid synthesis, and disrupt electron transport chains (Daglia 2012). Whether these specific mechanisms operate in our extracts requires mechanistic studies with isolated compounds.

The differential efficacy against gram‐positive versus gram‐negative bacteria reflects fundamental cell wall architecture differences. Gram‐positive bacteria possess thick peptidoglycan layers (20–80 nm) but lack the outer membrane present in gram‐negative species (Vaara 1992). This outer membrane, composed of lipopolysaccharides with negatively charged phosphate groups and fatty acid chains, functions as a permeability barrier against hydrophobic antimicrobial compounds (Vaara 1992). The relatively stronger activity against gram‐positive bacteria (15–20 mm zones) compared to gram‐negative species (10–14 mm zones) suggests that primary bioactive compounds are predominantly hydrophobic (capsaicinoids, flavonoid aglycones) or moderately polar (phenolic acids).

The notable activity against *V. cholerae* (22 mm at 125 μg/disc) and *E. coli* (20 mm) is significant given these pathogens' roles in waterborne and foodborne disease outbreaks. These findings support potential applications as natural food preservatives and antimicrobial agents (Tajkarimi et al. 2010). Capsaicinoids contribute to antibacterial activity through membrane potential disruption, ATP depletion (50%–70% reduction at 100 μg/mL), and reactive oxygen species generation (Freire‐Moran et al. 2011). Saponins enhance antibacterial efficacy through membrane permeabilization via cholesterol interaction and pore formation, with MIC values of 64–256 μg/mL against *S. aureus* and *E. coli* (Güçlü‐Üstündağ and Mazza 2007). The synergistic effects likely exceed individual activities, explaining the broad‐spectrum profile. Compared to other plant extracts, *C. frutescens* shows competitive activity: green tea demonstrates 12–18 mm zones against *S. aureus* at 100 μg/disc (Adelani‐Akande et al. 2015), pomegranate peel shows 15–20 mm at 200 μg/disc (Voravuthikunchai et al. 2004), and turmeric exhibits 10–15 mm at 100 μg/disc (Naz et al. 2007).

The 2.5‐fold difference in DPPH radical scavenging capacity between *C. frutescens* (IC_50_ = 111.96 μg/mL) and *C. annuum* (IC_50_ = 284.57 μg/mL) directly parallels their 3.5‐fold difference in total phenolic content, confirming phenolic compounds as primary determinants of antioxidant capacity. The DPPH assay measures the ability of antioxidants to donate hydrogen atoms or electrons to the stable DPPH radical, resulting in its reduction and characteristic color change from deep purple to pale yellow (Brand‐Williams et al. 1995). Antioxidant mechanisms operate through: (i) hydrogen atom transfer (HAT), where phenolic hydroxyl groups donate hydrogen atoms to DPPH radicals, forming stable phenoxyl radicals resonance‐stabilized through aromatic ring delocalization; (ii) single electron transfer (SET), where phenolic compounds transfer electrons to DPPH; and (iii) transition metal chelation, where phenolic compounds chelate pro‐oxidant metals (Fe^2+^, Cu^+^) through catechol groups, preventing Fenton reaction‐mediated hydroxyl radical generation (Taslimi and Gulçin 2018). This stabilization prevents propagation of free radical chain reactions that would cause oxidative damage to cellular macromolecules including lipids (lipid peroxidation), proteins (carbonyl formation), and DNA (8‐oxo‐deoxyguanosine, strand breaks) (Halliwell 2007).

The moderate activity relative to ascorbic acid (IC_50_ = 8.32 μg/mL) reflects structural differences between complex polyphenols and simple reducing agents. While ascorbic acid rapidly reduces DPPH through direct electron transfer (k = 10^5^ M^−1^s^−1^), polyphenolic compounds may require conformational changes or exhibit steric hindrance that reduces reaction kinetics (k = 10^2^–10^3^ M^−1^s^−1^) (Rice‐Evans et al. 1996). However, polyphenols offer advantages including lipophilicity enabling cell membrane penetration, multiple reactive sites providing sustained antioxidant capacity, and additional biological activities such as gene expression modulation (Nrf2 activation), pro‐oxidant enzyme inhibition (NADPH oxidase, xanthine oxidase), and enhancement of endogenous antioxidants (glutathione, catalase, SOD) (Halliwell 2007).

The DPPH scavenging IC_50_ values for *C. frutescens* (111.96 μg/mL) compare favorably with other recognized antioxidant sources: blueberry extract (IC_50_ = 125–180 μg/mL) (Kähkönen et al. 1999), grape seed extract (IC_50_ = 85–120 μg/mL) (Soobrattee et al. 2005), pomegranate peel (IC_50_ = 140–190 μg/mL) (Gözlekçi et al. 2011), green tea extract (IC_50_ = 65–95 μg/mL) (Gramza‐Michałowska et al. 2017), and turmeric extract (IC_50_ = 180–250 μg/mL) (Ak and Gülçin 2008). This positions *C. frutescens* within the upper tier of dietary antioxidant sources.

The practical implications extend to food preservation applications. Lipid peroxidation represents a major cause of food quality deterioration, particularly in lipid‐rich foods, producing off‐flavors, rancidity, and nutrient degradation (Shahidi and Zhong 2010). Natural antioxidants from *C. frutescens* could extend shelf‐life by 30%–50% based on peroxide value reduction, while meeting consumer demand for clean‐label products free from synthetic preservatives like BHA and BHT, which face regulatory restrictions due to potential carcinogenicity concerns (Scientific Opinion on the Re‐Evaluation of Butylated Hydroxytoluene BHT (E 321) as a Food Additive 2012).

From a health perspective, the antioxidant properties support protection against oxidative stress‐related pathologies. Chronic oxidative stress contributes to atherosclerosis through LDL oxidation, foam cell development, and endothelial dysfunction; neurodegenerative diseases (Alzheimer's, Parkinson's) via neuronal damage and protein aggregation; and carcinogenesis through DNA mutation, genomic instability, and promotion of proliferation through redox‐sensitive transcription factors (Halliwell 2007). Epidemiological studies show inverse associations between dietary antioxidant intake and chronic disease risk: 20%–30% reduction in cardiovascular disease mortality with high vs. low flavonoid intake (> 500 vs. < 150 mg/day) (Hertog et al. 1993), and 15%–25% reduction in cancer incidence with high vs. low polyphenol consumption (> 800 vs. < 400 mg/day) (Bobe et al. 2008). Regular consumption of antioxidant‐rich foods like *C. frutescens* (providing 15–20 mg GAE per 100 g fresh fruit) may reduce disease risk through neutralization of reactive oxygen species and enhancement of endogenous antioxidant defenses via Nrf2‐ARE pathway activation (Kensler et al. 2007a).

The remarkable protein denaturation inhibition demonstrated by both extracts ( *C. frutescens* : 99.57%; *C. annuum* : 99.42% at 250 μg/mL) provides mechanistic insight into their anti‐arthritic potential. Protein denaturation plays a central role in rheumatoid arthritis pathogenesis, where heat (synovial inflammation), acidosis (pH 6.5–7.0 in inflamed joints vs. 7.4 normal), and oxidative stress (10‐fold increased ROS in RA synovium) cause protein unfolding and aggregation (McInnes and Schett 2011). Denatured proteins become autoantigens triggering autoimmune responses characterized by autoantibody production (rheumatoid factor, anti‐CCP), complement activation, and chronic inflammation (IL‐1β, TNF‐α, IL‐6 elevation) that progressively destroys articular cartilage and bone (McInnes and Schett 2011).

The mechanism of protein stabilization likely involves: (i) hydrogen bonding, where phenolic hydroxyl groups and flavonoid carbonyl oxygens form hydrogen bonds with protein backbone amide groups, stabilizing secondary structure elements and increasing denaturation temperature (Tm) by 5°C–15°C; (ii) hydrophobic interactions, where aromatic rings establish hydrophobic interactions with nonpolar amino acid residues, maintaining protein tertiary structure; (iii) electrostatic interactions, where ionized phenolic groups interact with positively charged residues, contributing to conformational stability (Arts et al. 2001). These interactions increase the Gibbs free energy barrier for protein unfolding from −20 to −35 kcal/mol, thereby elevating thermal denaturation temperature and protecting proteins under stress conditions (Arts et al. 2001).

The comparable efficacy between *C. frutescens* (99.57%) and *C. annuum* (99.42%), and their similarity to diclofenac sodium (99.78%), is clinically significant. Diclofenac, a non‐selective COX‐1/COX‐2 inhibitor, represents a gold standard anti‐inflammatory drug (typical dose: 75–150 mg/day). However, diclofenac carries significant adverse effects: gastrointestinal (peptic ulcers in 15%–30% patients, bleeding in 1%–2% annual serious GI events), renal (acute kidney injury, chronic kidney disease, hypertension), cardiovascular (40% increased myocardial infarction risk, 20% increased stroke risk), and hepatotoxicity (transaminase elevation in 3%–5% patients) (McGettigan and Henry 2013; Sostres et al. 2010). The equivalent protein stabilization achieved by *Capsicum* extracts suggests potential for developing natural anti‐inflammatory therapeutics with improved safety profiles, particularly for chronic use in elderly patients with multiple comorbidities.

Beyond protein stabilization, capsaicinoids contribute through: (i) substance P depletion, where capsaicin depletes this 11‐amino acid neuropeptide mediating pain transmission and inflammatory responses through initial TRPV1 activation causing massive release, followed by prolonged receptor desensitization (hours–weeks) and reduced neuropeptide synthesis; (ii) pain relief, where clinical trials demonstrate capsaicin efficacy in OA (0.025%–0.075% topical cream, 4× daily) with 40%–50% pain reduction vs. 20% placebo (Derry et al. 2017; Deal et al. 1991); (iii) COX‐2 inhibition, where capsaicin and analogs inhibit COX‐2 expression through NF‐κB pathway suppression (IC_50_ = 5–15 μM), reducing prostaglandin E₂ production by 40%–60% without affecting COX‐1, suggesting selective anti‐inflammatory effects with reduced GI toxicity (Joe and Lokesh 1994).

*Capsicum* phytochemicals inhibit nuclear factor‐κB (NF‐κB) activation through: (i) IκB kinase (IKK) inhibition preventing IκB phosphorylation and degradation; (ii) direct NF‐κB DNA‐binding inhibition; (iii) antioxidant effects reducing ROS‐mediated NF‐κB activation (Chou 2006a). This reduces transcription of pro‐inflammatory cytokines including TNF‐α (50%–70% reduction), IL‐1β (40%–60% reduction), and IL‐6 (45%–65% reduction) (Chou 2006a). Additionally, flavonoids inhibit phospholipase A₂ (IC_50_ = 10–50 μM), reducing arachidonic acid release and subsequent leukotriene/prostaglandin synthesis (Kim et al. 2004). This multi‐target anti‐inflammatory profile supports traditional use in arthritis treatment and warrants investigation in relevant inflammatory disease models (adjuvant‐induced arthritis, collagen‐induced arthritis) to confirm in vivo efficacy, optimal dosing, and safety.

The significant cytotoxic activity of *C. frutescens* (LC_50_ = 29.24 μg/mL) meeting NCI preliminary screening criteria (LC_50_ < 30 μg/mL) represents an important finding warranting further investigation (Suffness and Pezzuto 1991). However, the brine shrimp lethality bioassay, while useful for preliminary screening, measures general toxicity rather than cancer‐specific effects. The correlation with human cancer cell cytotoxicity is moderate (*r* = 0.70–0.85) but imperfect (Carballo et al. 2002a; Meyer et al. 1982b).

The two‐fold difference in cytotoxic potency between *C. frutescens* (LC_50_ = 29.24 μg/mL) and *C. annuum* (LC_50_ = 59.37 μg/mL) likely reflects differences in bioactive compound profiles. Multiple mechanisms may contribute, though all remain speculative without compound identification: (i) potential capsaicinoid‐mediated apoptosis, (ii) possible phenolic compound cytotoxicity, (iii) hypothetical saponin‐mediated membrane disruption. Literature reports indicate that capsaicin induces apoptosis (Trachootham et al. 2009; Podolak et al. 2010). However, these mechanisms remain speculative without compound identification and mechanistic studies. To establish genuine anticancer potential, the following studies are essential: (i) cytotoxicity screening against panels of human cancer cell lines with selectivity determination, (ii) mechanistic studies (apoptosis markers, cell cycle analysis, caspase activation), (iii) identification of active compounds through bioassay‐guided fractionation, (iv) in vivo studies using xenograft tumor models, and (v) pharmacokinetic and toxicity assessments (Trachootham et al. 2009; Podolak et al. 2010; Bley et al. 2012).

The chemopreventive potential extends beyond direct cytotoxicity through: (i) antimutagenic effects, where antioxidant properties prevent oxidative DNA damage (8‐oxo‐dG formation reduced by 40%–60%) that initiates carcinogenesis; (ii) anti‐inflammatory effects, as reduction of chronic inflammation, a recognized cancer risk factor (15%–20% cancers inflammation‐associated), through NF‐κB inhibition and cytokine suppression (Mantovani et al. 2008); (iii) phase II enzyme induction, where phenolic compounds activate Nrf2‐ARE pathway, inducing detoxification enzymes (glutathione S‐transferase, quinone reductase, UDP‐glucuronosyltransferase) that facilitate carcinogen elimination (2–4 fold enzyme induction) (Kensler et al. 2007b); (iv) angiogenesis inhibition, where flavonoids and capsaicinoids inhibit VEGF expression and VEGF receptor signaling, reducing tumor neovascularization and limiting metastatic potential (Folkman 2007). The combination of antimutagenic, antiproliferative, anti‐angiogenic, and immune‐enhancing properties positions *Capsicum* extracts as promising candidates for both cancer prevention (dietary supplementation) and adjunctive cancer therapy (combination with chemotherapy). *While the nutritional profiles suggest potential for addressing micronutrient deficiencies, translation to actual health outcomes requires: (i) bioavailability studies to determine mineral absorption rates, (ii) intervention trials in deficient populations, and (iii) assessment of long‐term consumption effects on nutritional status markers*.

*C. frutescens*, with 2.5‐fold higher DPPH scavenging activity (IC_50_ = 111.96 μg/mL), 3.5‐fold elevated phenolic content (18.85 μg GAE/mg), enhanced antibacterial efficacy (inhibition zones: 1522 mm at 125 μg/disc), and significant cytotoxic activity meeting NCI preliminary criteria (LC_50_ = 29.24 μg/mL) shows potential for pharmaceutical development, functional foods targeting oxidative stress and inflammation, natural antimicrobial applications, and cancer chemoprevention research. However, these applications require: (i) identification of active compounds, (ii) mechanistic studies, (iii) in vivo validation, (iv) safety and toxicity assessments, (v) bioavailability studies, and (vi) clinical trials.

*C. annuum* , characterized by higher calcium (6.21 mg/100 g), phosphorus (5.78 mg/100 g), and dietary fiber (5.72%) alongside elevated flavonoid (37.8 g RE%) and saponin (18.6 g DE%) levels, offers advantages for bone health promotion (optimal Ca:P ratio 1.07:1), digestive wellness applications (prebiotic effects, glycemic control), and conditions where these specific phytochemical classes provide therapeutic benefits (immunomodulation, cholesterol management). Its comparable anti‐arthritic activity (99.42% protein denaturation inhibition, equivalent to diclofenac sodium) and moderate antibacterial and antioxidant properties support traditional medicinal uses and integration into health‐promoting dietary patterns.

The complementary properties suggest potential for synergistic formulations combining both species in functional food formulations (multi‐*Capsicum* blends, mixed powders), maximizing nutritional and bioactive compound delivery while optimizing health‐promoting effects. This approach would provide: comprehensive mineral profile (high Fe + K from *C. frutescens* + high Ca + *P* from *C. annuum* ), broad‐spectrum phytochemicals (phenolics + alkaloids + flavonoids + saponins), and multiple biological activities (antioxidant + antibacterial + anti‐inflammatory + cytotoxic). Future studies should test synergy using isobologram analysis and combination index determination (Chou 2006b).

The significant biological activities demonstrated by these readily cultivable, economically accessible botanical resources (growing season: 90–120 days, yield: 10–20 tons/ha, production cost: $0.50–$1.50/kg) have important implications for public health, particularly in resource‐limited settings where access to conventional pharmaceuticals remains constrained (only 50%–60% population access in low‐income countries) (WHO, 2021). Promotion of *Capsicum* cultivation and consumption could support nutritional security while providing accessible sources of bioactive compounds for disease prevention and health promotion, contributing to sustainable development goals (SDG 2: Zero Hunger, SDG 3: Good Health and Well‐being) (United Nations, 2015).

## 5 Study Limitations

Several important limitations warrant consideration. First, the use of absolute methanol as the sole extraction solvent, while standard for preliminary screening, may not extract the full range of bioactive compounds (Azmir et al. 2013). Aqueous methanol (70%–80%) or sequential extraction with solvents of increasing polarity would provide more comprehensive phytochemical profiles (Azmir et al. 2013). Extraction yield (8.7% for *C. frutescens* , 6.3% for *C. annuum* ) represents only a fraction of total plant material, with remaining compounds potentially bioactive. Second, the most significant limitation is the absence of individual compound identification via HPLC‐DAD‐MS/MS, LC‐QTOF‐MS, or GC–MS due to resource constraints. This prevents definitive attribution of observed biological activities to specific compounds, limits standardization potential for pharmaceutical development, and precludes structure–activity relationship studies (Materska and Perucka 2005; Barbero et al. 2006). While qualitative tests confirmed the presence of alkaloids, flavonoids, terpenoids, saponins, steroids, and tannins, and quantitative assays measured total phenolic, flavonoid, and saponin content, identification of individual compounds within each class remains incomplete. Future HPLC‐DAD‐MS/MS profiling is essential to characterize specific compound profiles and establish definitive structure–activity relationships. Third, all biological assays (DPPH, antibacterial, anti‐arthritic, cytotoxicity) were conducted in vitro and cannot predict in vivo efficacy due to pharmacokinetic factors (absorption, first‐pass metabolism, distribution, excretion), bioavailability limitations (polyphenols typically 2%–20% bioavailable), metabolic transformations (phase I/II metabolism), and complex physiological interactions. Fourth, the disc diffusion assay, while useful for preliminary antibacterial screening, does not provide quantitative potency measures (MIC/MBC values) or information on bacteriostatic versus bactericidal mechanisms. MIC (minimum inhibitory concentration) and MBC (minimum bactericidal concentration) determinations would be necessary to establish clinical relevance, enable dose–response characterization, and allow meaningful comparisons with standard antibiotics. Fifth, the brine shrimp lethality bioassay, while useful for preliminary toxicity screening and showing moderate correlation with mammalian cell cytotoxicity (r = 0.70–0.85) (Meyer et al. 1982a; Kensler et al. 2007a), measures general toxicity rather than cancer‐specific effects and provides no information on: (i) selectivity (cancer cells vs. normal cells), (ii) mechanism of action (apoptosis, necrosis, cell cycle arrest, autophagy), or (iii) relevant cancer biology endpoints (angiogenesis inhibition, metastasis suppression, drug resistance modulation). Validation through authenticated human cancer cell line panels is essential. Sixth, although all experiments included three biological replicates with triplicate technical measurements (*n* = 9 total), larger sample sizes and multiple harvest seasons would increase statistical power for detecting smaller effect sizes, reduce type II error risk, and account for seasonal/geographical variations in phytochemical content.

## 6 Conclusion

This comparative investigation establishes *Capsicum frutescens* and *Capsicum annuum* as valuable botanical resources with distinct yet complementary nutritional and bioactive profiles through the first comprehensive integrated analysis using standardized methodologies. The species‐specific advantages identified—with *C. frutescens* demonstrating superior antioxidant capacity (2.5‐fold higher, *p* < 0.001), correlating with 3.5‐fold greater phenolic content and exhibiting significant cytotoxic potential in preliminary screening meeting National Cancer Institute criteria, though validation in human cancer cell lines and in vivo models is necessary to confirm anticancer applications. Additionally, it provides elevated iron (4.91), potassium (5.21), and zinc (0.20 mg/100 g), positioning it as particularly valuable for addressing micronutrient deficiencies in developing regions (Aburto, Ziolkovska, et al. 2013b). Conversely, *C. annuum* offers advantages through higher calcium (6.21), phosphorus (5.78), and dietary fiber (5.72 mg/100 g) with an optimal Ca:P ratio (1.07:1) beneficial for bone health, alongside elevated flavonoids and saponins supporting digestive wellness, glycemic control, and cardiovascular health. Remarkably, both species exhibited equivalent anti‐arthritic activity (approximately 99% protein denaturation inhibition), comparable to diclofenac sodium, validating their traditional use in treating inflammatory conditions. These species‐specific advantages support targeted applications: *C. frutescens* excels in antioxidant‐rich functional foods, natural antimicrobials, and pharmaceutical development, while *C. annuum* suits formulations targeting bone health and metabolic wellness. The complementary profiles suggest promising opportunities for synergistic combinations. Beyond health‐promoting properties, these readily cultivable crops hold significant potential for addressing public health challenges in resource‐limited settings, supporting nutritional security and contributing to sustainable development goals. Future research should prioritize advanced phytochemical profiling via HPLC‐DAD‐MS/MS for compound identification, bioassay‐guided fractionation to isolate bioactive compounds, mechanistic investigations elucidating molecular targets, and in vivo validation using animal disease models. Additionally, comprehensive pharmacokinetic studies, MIC/MBC determination for antibacterial standardization, clinical trials, and standardization protocol development are essential. Systematically addressing these priorities will transform the promising bioactivities into evidence‐based therapeutic interventions and functional food products to contribute meaningfully to global human health and nutritional security.

## Author Contributions

**Muhammad Mamunur Rashid Mahib:** writing – original draft, writing – review and editing, visualization, formal analysis, software, validation, data curation.

## Funding

This work was supported by the Research and Publication Cell, University of Chittagong (Grant no.: 41/2023) and by the Ministry of Science and Technology, Bangladesh (SRG‐241209, 2024–2025) to M.G.K.

## Conflicts of Interest

The authors declare no conflicts of interest.

## Supporting information


**Figure S1:** Schematic representation of the collection and extraction processes for *Capsicum frutescens* and *Capsicum annuum* fruits. This figure shows the extraction workflow for *Capsicum frutescens* and *Capsicum annuum* fruits. Fresh fruits are collected, washed, and air‐dried for 15 days at 25°C, then ground into powder. The powder undergoes solvent extraction using methanol, n‐butanol, n‐hexane, and chloroform for 7 days with intermittent shaking. Extracts are filtered, concentrated by rotary evaporation at 40°C under vacuum, and stored in amber glass bottles at 4°C.
**Figure S2:** Schematic of the AOAC‐based methods used for the proximate and mineral analysis of for *Capsicum frutescens* and *Capsicum annuum* fruits. This figure outlines the AOAC‐based analytical methods for proximate and mineral analysis of *Capsicum frutescens* and *Capsicum annuum* fruits. After collection, fruits are cleaned, air‐dried, and ground into powder. The material is then sub‐sampled for four parallel analyses: moisture content determination (drying at 105°C until constant weight), ash content analysis (muffle furnace at 600°C for 6 h to determine total mineral content), crude protein quantification (Micro‐Kjeldahl method using H₂SO₄ distillation and titration), and lipids, crude fiber, and mineral analysis (using Bligh & Dyer method, acid/alkali digestion, and AAS/Flame Photometry). All methods follow AOAC standard protocols.
**Figure S3:** Flowchart depicting the pharmacological evaluation of *Capsicum frutescens* and *Capsicum annuum* fruits extracts through various assays. This figure illustrates the pharmacological evaluation framework for *Capsicum frutescens* and *Capsicum annuum* fruit extracts across four bioactivity assays. Antioxidant activity is assessed using the DPPH free radical scavenging assay (10–1000 μg/mL, 30‐min dark incubation, absorbance at 517 nm, with ascorbic acid as control). Cytotoxicity is evaluated via brine shrimp lethality assay (10–1000 μg/mL concentrations, 24‐h exposure, LC50 determined by Probit analysis). Antibacterial activity is tested using disc diffusion method against Gram‐positive and Gram‐negative bacteria on Mueller‐Hinton agar (37°C, 24 h), with ciprofloxacin as reference. Anti‐arthritic activity is measured through protein denaturation inhibition test using bovine serum albumin (incubation at 37°C for 30 min, heating at 70°C for 10 min, absorbance at 660 nm).

## Data Availability

The data that support the findings of this study are available from the corresponding author upon reasonable request.
