# Supplementary material for: Comparative Profiling of Capsicum frutescens and C. annuum Reveals Superior Bioactivities and Nutritional Advantages for Functional Food Applications
Source: Food Sci Nutr. 2026 Jan 9;14(1):e71426. doi: 10.1002/fsn3.71426 (PMC12789648; doi:10.1002/fsn3.71426)
Supplement: Supplementary file 1 — Figure S1: Schematic representation of the collection and extraction processes for Capsicum frutescens and Capsicum annuum fruits. This figure shows the extraction workflow for Capsicum frutescens and Capsicum annuum fruits. Fresh fruits are collected, washed, and air‐dried for 15 days at 25°C, then ground into powder. The powder undergoes solvent extraction using methanol, n‐butanol, n‐hexane, and chloroform for 7 days with intermittent shaking. Extracts are filtered, concentrated by rotary evaporation at 40°C under vacuum, and stored in amber glass bottles at 4°C. Figure S2: Schematic of the AOAC‐based methods used for the proximate and mineral analysis of for Capsicum frutescens and Capsicum annuum fruits. This figure outlines the AOAC‐based analytical methods for proximate and mineral analysis of Capsicum frutescens and Capsicum annuum fruits. After collection, fruits are cleaned, air‐dried, and ground into powder. The material is then sub‐sampled for four parallel analyses: moisture content determination (drying at 105°C until constant weight), ash content analysis (muffle furnace at 600°C for 6 h to determine total mineral content), crude protein quantification (Micro‐Kjeldahl method using H₂SO₄ distillation and titration), and lipids, crude fiber, and mineral analysis (using Bligh & Dyer method, acid/alkali digestion, and AAS/Flame Photometry). All methods follow AOAC standard protocols. Figure S3: Flowchart depicting the pharmacological evaluation of Capsicum frutescens and Capsicum annuum fruits extracts through various assays. This figure illustrates the pharmacological evaluation framework for Capsicum frutescens and Capsicum annuum fruit extracts across four bioactivity assays. Antioxidant activity is assessed using the DPPH free radical scavenging assay (10–1000 μg/mL, 30‐min dark incubation, absorbance at 517 nm, with ascorbic acid as control). Cytotoxicity is evaluated via brine shrimp lethality assay (10–1000 μg/mL concentrations, 24‐h exposure, LC50 [file FSN3-14-e71426-s001.pptx]

## Slide 1
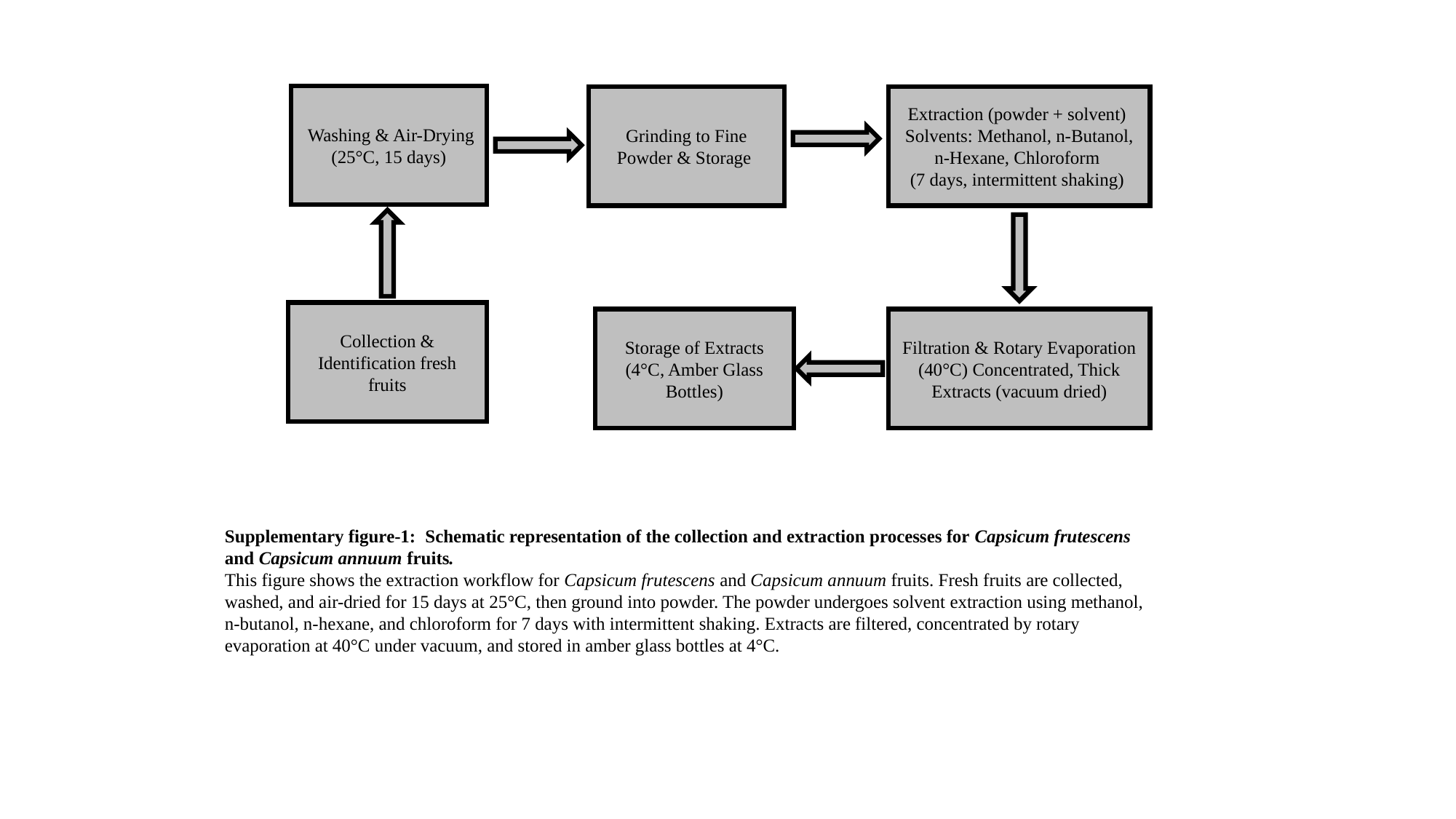

Washing & Air-Drying (25°C, 15 days)
Extraction (powder + solvent)
Solvents: Methanol, n-Butanol, n-Hexane, Chloroform
(7 days, intermittent shaking)
Grinding to Fine Powder & Storage
Collection & Identification fresh fruits
Storage of Extracts (4°C, Amber Glass Bottles)
Filtration & Rotary Evaporation (40°C) Concentrated, Thick Extracts (vacuum dried)
Supplementary figure-1: Schematic representation of the collection and extraction processes for Capsicum frutescens and Capsicum annuum fruits.
This figure shows the extraction workflow for Capsicum frutescens and Capsicum annuum fruits. Fresh fruits are collected, washed, and air-dried for 15 days at 25°C, then ground into powder. The powder undergoes solvent extraction using methanol, n-butanol, n-hexane, and chloroform for 7 days with intermittent shaking. Extracts are filtered, concentrated by rotary evaporation at 40°C under vacuum, and stored in amber glass bottles at 4°C.

## Slide 2
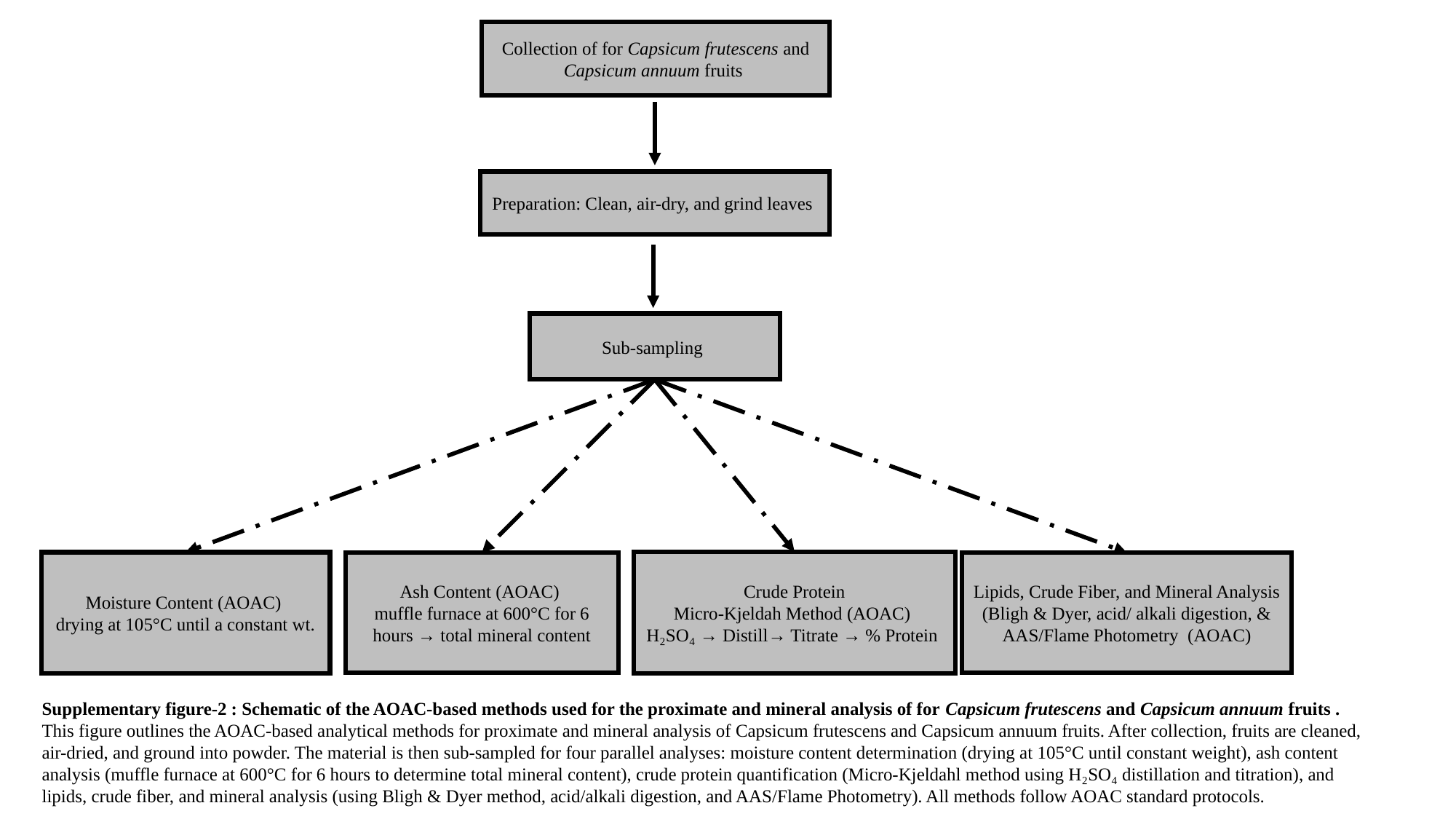

Collection of for Capsicum frutescens and Capsicum annuum fruits
Preparation: Clean, air-dry, and grind leaves
Sub-sampling
Crude Protein
Micro-Kjeldah Method (AOAC)
H₂SO₄ → Distill→ Titrate → % Protein
Moisture Content (AOAC)
drying at 105°C until a constant wt.
Ash Content (AOAC)
muffle furnace at 600°C for 6 hours → total mineral content
Lipids, Crude Fiber, and Mineral Analysis
(Bligh & Dyer, acid/ alkali digestion, & AAS/Flame Photometry (AOAC)
Supplementary figure-2 : Schematic of the AOAC-based methods used for the proximate and mineral analysis of for Capsicum frutescens and Capsicum annuum fruits .
This figure outlines the AOAC-based analytical methods for proximate and mineral analysis of Capsicum frutescens and Capsicum annuum fruits. After collection, fruits are cleaned, air-dried, and ground into powder. The material is then sub-sampled for four parallel analyses: moisture content determination (drying at 105°C until constant weight), ash content analysis (muffle furnace at 600°C for 6 hours to determine total mineral content), crude protein quantification (Micro-Kjeldahl method using H₂SO₄ distillation and titration), and lipids, crude fiber, and mineral analysis (using Bligh & Dyer method, acid/alkali digestion, and AAS/Flame Photometry). All methods follow AOAC standard protocols.

## Slide 3
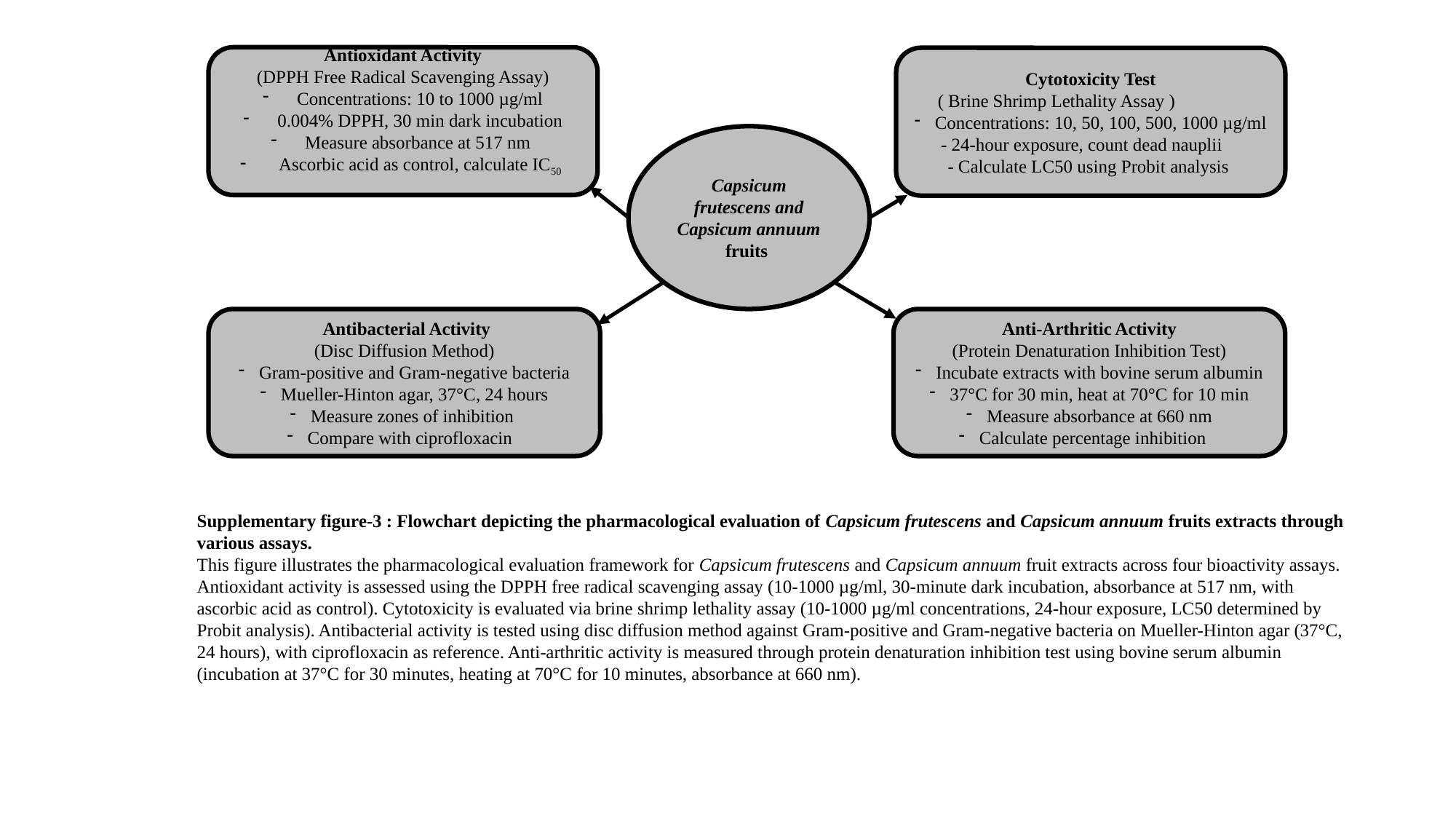

Antioxidant Activity
(DPPH Free Radical Scavenging Assay)
Concentrations: 10 to 1000 µg/ml
0.004% DPPH, 30 min dark incubation
Measure absorbance at 517 nm
 Ascorbic acid as control, calculate IC50
Cytotoxicity Test
( Brine Shrimp Lethality Assay )
Concentrations: 10, 50, 100, 500, 1000 µg/ml
- 24-hour exposure, count dead nauplii
- Calculate LC50 using Probit analysis
Capsicum frutescens and Capsicum annuum fruits
 Antibacterial Activity
(Disc Diffusion Method)
Gram-positive and Gram-negative bacteria
Mueller-Hinton agar, 37°C, 24 hours
Measure zones of inhibition
Compare with ciprofloxacin
Anti-Arthritic Activity
(Protein Denaturation Inhibition Test)
Incubate extracts with bovine serum albumin
37°C for 30 min, heat at 70°C for 10 min
Measure absorbance at 660 nm
Calculate percentage inhibition
Supplementary figure-3 : Flowchart depicting the pharmacological evaluation of Capsicum frutescens and Capsicum annuum fruits extracts through various assays.
This figure illustrates the pharmacological evaluation framework for Capsicum frutescens and Capsicum annuum fruit extracts across four bioactivity assays. Antioxidant activity is assessed using the DPPH free radical scavenging assay (10-1000 µg/ml, 30-minute dark incubation, absorbance at 517 nm, with ascorbic acid as control). Cytotoxicity is evaluated via brine shrimp lethality assay (10-1000 µg/ml concentrations, 24-hour exposure, LC50 determined by Probit analysis). Antibacterial activity is tested using disc diffusion method against Gram-positive and Gram-negative bacteria on Mueller-Hinton agar (37°C, 24 hours), with ciprofloxacin as reference. Anti-arthritic activity is measured through protein denaturation inhibition test using bovine serum albumin (incubation at 37°C for 30 minutes, heating at 70°C for 10 minutes, absorbance at 660 nm).
